# From CAR-T Cells to Exosome-Based Immunotherapy: Exploring the Frontiers of Cell-Free Targeted Cancer Therapeutics

**DOI:** 10.3390/cells15010070

**Published:** 2025-12-31

**Authors:** Alexandru Tîrziu, Florina Maria Bojin, Oana Isabella Gavriliuc, Cosmin Ioan Faur, Virgil Păunescu

**Affiliations:** 1 Department of Functional Sciences, “Victor Babes” University of Medicine and Pharmacy, Tudor Vladimirescu Street, No. 14, 300174 Timisoara, Romania; alexandru.tirziu@umft.ro (A.T.); gavriliuc.oana@umft.ro (O.I.G.); vpaunescu@umft.ro (V.P.); 2 Center for Gene and Cellular Therapies in the Treatment of Cancer Timisoara-OncoGen, Clinical Emergency County Hospital “Pius Brinzeu” Timisoara, No. 156 Liviu Rebreanu, 300723 Timisoara, Romania; 3 Immuno-Physiology and Biotechnologies Center, Department of Functional Sciences, “Victor Babes” University of Medicine and Pharmacy, No. 2 Eftimie Murgu Square, 300041 Timisoara, Romania; 4 Department of Orthopedics—Traumatology, Urology, and Medical Imaging, “Victor Babes” University of Medicine and Pharmacy, Eftimie Murgu Square, No. 2, 300041 Timisoara, Romania; faur.cosmin@umft.ro

**Keywords:** CAR-T cell therapy, CAR-NK cells, exosome-based immunotherapy, cancer immunotherapy, cell-free therapeutics, tumor microenvironment, extracellular vesicles

## Abstract

Chimeric antigen receptor (CAR) cell therapies have revolutionized cancer immunotherapy by enabling targeted and potent antitumor immune responses. However, clinical challenges such as limited efficacy in solid tumors, severe toxicities including cytokine release syndrome (CRS), and manufacturing complexities restrict their broader use. Recently, CAR cell-derived exosomes (CAR-Exos) have emerged as promising cell-free therapeutic alternatives that retain the key antitumor functionalities of their parent cells while potentially overcoming the limitations of live cellular therapies. These nanoscale vesicles can deliver bioactive CAR molecules, cytotoxic proteins, and immunomodulatory cargo, enabling targeted tumor cell killing with reduced systemic toxicity and offering “off-the-shelf” applicability. This review comprehensively explores the biology, engineering, and therapeutic potential of CAR-Exos derived from T cells, natural killer (NK) cells, and other immune effectors. We discuss advances in isolation, characterization, and cargo profiling techniques, as well as preclinical and early clinical data supporting their application. Further, we address translational challenges including large-scale production, biodistribution, and immune evasion in tumor microenvironments. Combining cellular and exosomal CAR platforms holds promise to enhance efficacy and safety in cancer treatment, representing a frontier in targeted immunotherapy.

## 1. Introduction

CAR-T cell-derived exosomes represent an emerging approach in cancer immunotherapy. These nanoscale vesicles retain the targeting specificity of parent cells while offering potential advantages in safety and manufacturing scalability. However, translational challenges, including limited in vivo persistence, suboptimal tumor accumulation, and manufacturing standardization, require resolution before clinical implementation.

Since the beginning of CAR-T cell therapy in the late 1980s, significant advancements have been made, evolving from first-generation CARs to complex constructs with enhanced efficacy and persistence [1,2]. Despite remarkable success in hematologic cancers, challenges such as tumor recurrence, immunosuppressive tumor microenvironments, and severe toxicities have limited broader clinical application [3,4]. Moreover, the major limitations of CAR-T and CAR-NK therapies in solid tumors, like limited efficacy and increased toxicity risk, diminish their therapeutic effectiveness [5]. Therefore, there is an increased need for other engineering strategies such as TCR-T, STAR-T, or CAR-exosomes, to overcome the limitations of CAR cellular therapies on solid tumors.

Exosomes, nanoscale extracellular vesicles secreted by immune cells including CAR-T cells, have gained attention for their role in intercellular communication and as potential cell-free therapeutic agents [6,7]. Their ability to carry bioactive molecules and traverse biological barriers makes them promising tools to enhance targeted cancer immunotherapy [8,9].

CAR-exosomes (CAR-Exos) offer potential advantages as off-the-shelf therapeutic platforms. Their nanoscale size (30–150 nm) may enable enhanced tissue penetration compared to living cells, while surface-expressed CARs maintain tumor antigen specificity. Their non-replicative nature may reduce risks of cytokine release syndrome and neurotoxicity. However, this same property limits in vivo persistence, potentially requiring repeated administration for sustained antitumor activity.

CAR-T Exos retain cytotoxic activity comparable to parental cells and can modulate diverse cellular processes via their cargo of proteins and nucleic acids, making them a promising alternative for targeted cancer immunotherapy.

While CAR-T Exos have demonstrated cytotoxic activity and lower toxicity profiles in preclinical models, a comprehensive understanding of their functional characteristics, mechanisms of action, and clinical translation remains incomplete [3,10,11]. Controversies exist regarding the extent to which CAR-Exos can overcome immunosuppressive factors such as tumor-derived extracellular vesicles [12]. Moreover, insufficient characterization of CAR-Exos’ cargo, biodistribution, and large-scale production challenges delay their clinical development [4,12,13].

Despite promising preclinical data, CAR-exosomes face translational challenges distinct from living CAR cell therapies. Rapid hepatosplenic clearance limits tumor accumulation, variable cargo composition complicates manufacturing standardization, and the absence of in vivo expansion may restrict long-term efficacy. Critical comparative analyses of their efficacy, durability, and mechanistic equivalence to parent cells remain limited. This review addresses these gaps by assessing CAR-exosome biology, therapeutic potential, and translational barriers.

## 2. Chimeric Antigen Receptor Therapy

### 2.1. CAR Structure and Evolution of CAR Generations

Chimeric antigen receptors are engineered synthetic receptors that recognize and bind specific antigens and produce immune cell activation, independent of the regulatory mechanisms involved in immune synapse formation [14]. CARs comprise three mandatory domains to be functionally active: (1) an extracellular target-binding domain, derived from the single chain variable fragment (scFv) of a monoclonal antibody; (2) a transmembrane domain derived from immune synapse effector molecules; (3) an intracellular signaling domain; usually the intracellular domain is derived from CD3ζ or γ-chain of the high-affinity IgE Fc receptor [15].

First-generation CARs exerted potent cytotoxic responses but suffered from insufficient IL-2 production and short cell lifespan [16]. Second- and third-generation CARs addressed these limitations by incorporating co-stimulatory domains (4-1BB, ICOS, OX40 for T cells [17], and NKG2D, 2B4, DNAM-1 for NK cells [18]) fused to the intracellular signaling domain, enhancing persistence and functionality [19]. Fourth-generation CARs (TRUCKs) incorporate an NFAT-responsive element that upregulates cytokine production (IL-12, IL-13, GM-CSF) upon stimulation [16,20], while fifth-generation CARs include a cytokine receptor intracellular domain (IL-2Rβ) that activates JAK-STAT and MAPK pathways, improving proliferation, survival, and activation [21,22].

CAR-T therapy’s autologous nature poses challenges for heavily pre-treated patients with depleted T lymphocyte count [23], while allogeneic administration risks graft-versus-host disease. Current efforts focus on “off-the-shelf” universal CAR-T cells with HLA class I and/or TCR gene knockdown using TALEN, zinc finger nucleases, or CRISPR/Cas9, minimizing graft-versus-host disease risk while maintaining cytotoxic function [24,25].

CAR-T cells cannot recognize intracellular tumor-specific neoantigens, targeting only extracellular TAAs [26], which are often expressed on healthy tissues, causing “off-target” toxicity. Strategies targeting MHC-I-bound epitopes leverage the MHC class I pathway’s presentation of intracellular proteins undergoing proteasomal cleavage, expanding the T cell recognition repertoire [27]. However, both CAR-T cells and tumor-infiltrating lymphocytes face similar TME penetration challenges.

TCR-T cell therapy engineers T cells to express novel TCR α/β variable chains recognizing tumor neoantigens, allowing intracellular antigen recognition beyond CAR-T’s surface protein targets. However, MHC class I dependence introduces complexity, requiring patient-specific MHC allele compatibility and precise matching, making this cell therapy more personalized than MHC-independent CAR-T cells [27]. Additionally, tumor cells may downregulate MHC class I molecules, rendering this approach ineffective, as demonstrated in anti-TP53 TCR-T breast cancer trials where time-limited responses correlated with MHC-I loss [28].

TCR- and TCR-like CAR-T cells enable efficient T cell activation by replacing the CAR scFv antigen-recognition domain with TCR or TCR-like antibody fragments, resulting in a TCR-dependent cytotoxic response. Compared to TCR-T cell therapy, this approach presents a significant advantage in terms of activation potential, as the co-stimulatory domains of this construct allow for a more vigorous activation while recognizing tumor neoantigens. Preclinical studies have shown promising results for in vitro and in vivo models of acute myeloid leukemia [29,30] and melanoma [31].

STAR-T (Synthetic T cell receptor and Antigen Receptor T) and TCAR-T cells are engineered T cells that replace the TCR α/β variable domains with antibody-derived variable regions so that the chimeric receptor becomes a functional part of the endogenous TCR/CD3 complex. Upon antigen engagement, the signal is transmitted via the native TCR molecules, allowing strong activation with less early dysfunction and improved proliferation compared to conventional CAR structures [32]. STAR-T cells mediate recognition in an MHC-independent, antibody-specific manner but signal via the intact TCR complex, effectively combining antibody specificity with native TCR signaling machinery. The double-chain design (separate α and β chains fused to antibody variable regions) prevents tonic signaling, which is attributed to classical CAR-T cell exhaustion [33].

T-CAR comprises a double-chain TCR αβ-based receptor with two scFv fragments in different configurations. Incorporating scFv domains into the natural CD3 complex, without additional costimulatory domains, leads to strong and sensitized responses against antigens. Compared to second or third-generation CARs, T-CAR-T cells exhibit potent antitumor responses with lower proliferation rates in the absence of costimulation (CD28, 4-1BB), potentially reducing on-target/off-tumor toxicities. Costimulation using RNA-LPX enhances T-CAR efficacy and persistence [34]. T-CAR’s intrinsic sensitivity could be beneficial for treating solid tumors with low antigen expression and hematologic malignancies [35].

The differentiation state of T cells used for CAR-T manufacturing critically determines therapeutic outcomes. T cells exist along a differentiation continuum from naïve cells through stem cell memory T cells (T_SCM_), central memory T cells (T_CM_), effector memory T cells (T_EM_), to terminally differentiated effector T cells (T_Eff_), with each stage exhibiting distinct functional properties [17,36].

These subsets are distinguished by characteristic surface markers: naïve and T_SCM_ cells express CD45RA, CCR7, CD62L, CD27, and CD28, with T_SCM_ additionally expressing CD95. T_CM_ cells are CD45RO+CCR7+CD62L+, while T_EM_ and T_Eff_ lose lymphoid homing markers (CCR7, CD62L) and progressively lose CD27 and CD28 expression [17,37]. Less differentiated subsets (naïve, T_SCM_, T_CM_) exhibit superior self-renewal capacity, proliferative potential, and long-term persistence, relying on oxidative phosphorylation for metabolic fitness. In contrast, T_EM_ and T_Eff_ cells demonstrate high immediate cytotoxicity but limited longevity, utilizing glycolytic metabolism that predisposes them to exhaustion [36,38,39].

The molecular basis for these differences involves transcription factors such as TCF-1 and LEF-1, which maintain stem-like memory programs, while T-BET and BLIMP1 drive terminal differentiation [39,40]. IL-7 and IL-15 support memory cell survival and are increasingly incorporated into manufacturing protocols [41,42].

CAR-T products enriched for less differentiated subsets demonstrate superior in vivo expansion, persistence, and tumor control in preclinical models [42,43]. T_STEM_-like CAR-T cells exhibited enhanced proliferation, sustained cytokine production, and improved antitumor responses compared to conventional products, even during chronic CAR stimulation in vitro. These responses depended on the presence of CD4+ T cells during production. The antitumor activity was enhanced by the co-administration of checkpoint inhibitor therapy [43]. Manufacturing approaches that preserve early memory phenotypes through IL-7/IL-15 supplementation or shortened culture duration increase T_SCM_/T_CM_ proportions and enhance therapeutic efficacy [41,42]. CAR-T cells produced from preselected naïve/stem memory T cells (CAR-_TN/SCM_) displayed superior antitumor activity compared to conventional CAR-T populations (CAR-T_BULK_) and were effective in counteracting leukemia rechallenge in humanized mice models of leukemia. They featured increased expansion rates and persistence, comparable transduction efficiency to CAR-T_BULK_ cells, less exhaustion, and were less prone to induce severe cytokine release syndrome [42].

### 2.2. CAR Engineering Methods

CAR-expressing cells are produced using various gene transfer strategies that enable CAR protein synthesis and membrane integration. An ideal gene transfer method should exhibit low immunogenicity, large cargo capacity, and sustained transgene expression [44]. Multiple types of gene transfection strategies have been studied. The most popular ones are the viral vectors (retro- and adenoviruses), transposons, mRNA/DNA transfection, and gene editing tools such as CRISPR/Cas9, TALENs (transcription activator-like effector nucleases), or ZFNs (zinc finger nucleases) [45].

#### 2.2.1. Retroviral Vectors

Retroviral vectors, particularly lentiviruses, are the most established CAR gene delivery method. Lentiviruses integrate transgenes into host chromosomes via reverse transcription, enabling stable, long-term CAR expression [44,45]. Unlike γ-retroviruses, lentiviruses can transduce non-dividing cells by nuclear pore translocation [46]. Third-generation lentiviral systems incorporate safety features including the deletion of accessory genes and self-inactivating LTRs to minimize insertional mutagenesis risk [47]. However, retroviral transduction shows low efficiency in NK cells (<15%) due to the limited LDL-receptor expression and innate antiviral responses [48]. These limitations have motivated the development of non-viral alternatives. Furthermore, differences in plasmid composition during lentiviral assembly can produce batch-to-batch variability [49]. Given these aspects, another direction for genetic material transfer is the use of non-viral vectors, particularly transposons, CRISPR-Cas9, zinc finger nucleases, and TALENs [50].

#### 2.2.2. Non-Viral Methods

##### Transposons

Transposons use “cut-and-paste” mechanisms for genomic integration. They present several advantages, including large cargo load, high transduction efficiency, ease of use, limited immunogenicity, and low production costs [51]. Sleeping Beauty (SB) and PiggyBac (PB) systems differ in target specificity and cargo capacity (PB: >100 kb; SB: <20 kb) [51,52]. Both can be delivered as plasmid DNA, mRNA, or purified protein [53], with mRNA delivery providing transient expression to limit genomic instability [54].

The hyperactive SB100X variant has demonstrated successful multi-gene transfer into human T cells via single electroporation procedures, generating CAR-T cells with enhanced specificity. Clinical trials using SB transposons have demonstrated >90% CAR-positive rates with favorable safety profiles without evidence of genotoxicity or malignant transformation [55], though the CARTELL trial reported lymphoma development in 2/10 patients, potentially linked to multiple integration events [56].

##### CRISPR/Cas9

CRISPR/Cas9 enables precise CAR integration into specific genomic loci via homology-directed repair (HDR) [57]. Enhancing HDR efficiency through cell cycle modulation (CDC7 inhibition) or NHEJ pathway suppression (DNA-PK inhibition) improves knock-in rates to 43–46% [58]. AAV-6 provides efficient delivery but has limited cargo capacity (<5 kb) [59]. Linearized or plasmid DNA delivery causes high cell toxicity and low knock-in efficiency because DSBs activate p53-mediated DNA damage pathways. Furthermore, NHEJ competes with HDR for DNA integration, reducing the desired knock-in rate [60]. Site-specific integration (e.g., into PDCD1 locus) enables simultaneous CAR expression and checkpoint inhibitor downregulation, improving antitumor efficacy [61].

**Figure 1:**
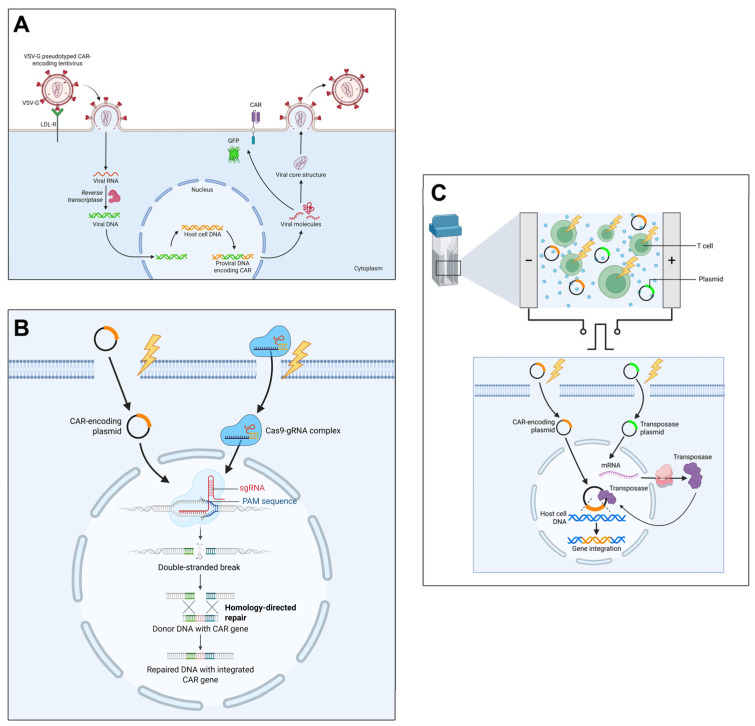
Overview of main cell transfection strategies for the generation of CAR-T cells. (**A**) Viral transduction: T cells are transduced with CAR-encoding lentiviral vectors, followed by reverse transcription and integration of the CAR transgene into host cell DNA, resulting in stable CAR expression. Created in BioRender. Tirziu, A. (2025), https://BioRender.com/lc2q988 (accessed on 29 December 2025); (**B**) CRISPR-Cas9 gene editing: T cells are electroporated with CAR-encoding plasmids and Cas9-gRNA complexes, enabling targeted double-stranded breaks and homology-directed repair, permitting precise CAR gene integration at desired loci. Created in BioRender. Tirziu, A. (2025), https://BioRender.com/y2bwtcj (accessed on 29 December 2025); (**C**) Non-viral transfection using transposons: T cells are electroporated with CAR-encoding and transposase plasmids; the transposase mediates CAR gene integration into host genome via transposition mechanisms, leading to stable CAR expression. Created in BioRender. Tirziu, A. (2025), https://BioRender.com/4ps78tl (accessed on 29 December 2025).

The main CAR cell engineering methods are summarized in Figure 1.

##### CAR-mRNA for Transient CAR Expression

Transient CAR expression might offer several advantages compared to stable gene integration in terms of mitigating the risk of insertional mutagenesis while reducing the toxicity profile. CAR expression arising from transient mRNA-encoded CARs results in a peak of CAR-positive effector cells that diminishes over time as CAR mRNA undergoes degradation and CAR proteins turn over. This constrained expression reduces the likelihood and magnitude of on-target/off-tumor toxicities driven by persistent CAR signaling, but may require repeated administration to maintain antitumor activity. Transient CAR expression can be achieved using mRNA electroporation or lipid nanoparticle (LNP)-coated mRNA [62]. The encoding mRNA is created through in vitro transcription (IVT) of a linear DNA template produced via PCR, followed by polyadenylation. Chemical modifications, such as N1-methyl-pseudouridine (M1ψ), improve protein expression, while reducing immunogenicity [63]. Kitte et al. reported that electroporation yields faster and higher initial CAR mRNA expression but is also associated with higher cytotoxicity and a greater fraction of exhausted T cells. The proportion of CAR-positive cells was comparable between mRNA and LV-CAR-T cells for the first three days post-transfection, after which mRNA-based CAR-T cells displayed a significantly lower percentage of CAR-positive cells. The evaluation of the cytotoxicity of mRNA-CAR-T cells compared to LV-CAR-T cells against different target cells and non-target cells showed non-significant differences in performance during the first two days post-transfection. These results demonstrate that while LNP-CAR-T cells exhibit a transient expression profile, their functional effects are comparable to those of lentivirus-mediated CAR expression. Furthermore, LNPs were also effective in CAR-NK generation [62,63]. Golubovskaya et al. reported that CD19-CAR and BCMA-CAR mRNA embedded in LNPs achieved high CAR expression, elevated levels of interferon-γ and granzyme B, and high cytotoxic profiles in primary NK cells. These findings highlight the potential of CAR-NK cells as allogeneic cancer immunotherapy [64].

Another feasible alternative to ex vivo CAR-T cell engineering is to generate CAR/TCR-T cells in situ by delivering poly-β-amino ester (PBAE) nanocarriers in complex with in vitro-transcribed antigen-specific CAR/TCR mRNAs. Parayath et al. demonstrated that these nanocarriers, through repeated infusions in murine models of leukemia, prostate cancer, and hepatitis B-induced hepatocellular carcinoma, successfully induce host T cells expressing tumor-specific CARs or virus-specific TCRs, leading to disease regression comparable to ex vivo engineered lymphocytes [65].

The main CAR engineering methods are described and compared in Table 1.

### 2.3. Clinical Aspects and Limitations of CAR Cells in Malignancies

CAR-T therapies achieve durable remissions in hematologic malignancies, with six FDA-approved products for B cell leukemia, lymphoma, and multiple myeloma [66]. CAR-T therapies achieve durable remissions in relapsed/refractory B cell malignancies, with response rates exceeding 80% in pediatric and adult acute lymphoblastic leukemia, demonstrating long-term survival benefits [67]. However, clinical applications are limited by severe toxicities and reduced efficacy in solid tumors [68].

Cytokine release syndrome (CRS) is one of the most feared complications of CAR-T cell therapy. It results from massive cytokine release (IL-6, IFN-γ, TNF-α) following CAR-T activation, manifesting as fever, hypotension, and multi-organ dysfunction [69,70]. Grade 3–4 CRS occurs in 10–20% of patients. Management includes tocilizumab (IL-6 receptor blockade) and corticosteroids [71]. For refractory cases or when tocilizumab is unavailable, corticosteroids—particularly dexamethasone—are used due to the blood–brain barrier penetration, though concerns exist regarding the potential impacts on therapeutic efficacy [72].

Immune effector cell-associated neurotoxicity syndrome (ICANS) affects 20–40% of patients, presenting as confusion, seizures, or cerebral edema [71]. Mechanisms include blood–brain barrier disruption and endothelial activation. Some brain mural cells express CD19, representing potential off-tumor targets [73].

Additional toxicities include tumor lysis syndrome (particularly in high tumor burden), B cell aplasia requiring immunoglobulin replacement, and prolonged cytopenias [74,75].

Solid tumor challenges include limited CAR-T infiltration, immunosuppressive tumor microenvironments, antigen heterogeneity, and on-target/off-tumor toxicity [76]. These persistent limitations have motivated the exploration of alternative approaches, including CAR-derived exosomes that may offer improved safety profiles while maintaining antitumor activity.

### 2.4. The Tumor Microenvironment (TME)

Chimeric antigen receptor T cell (CAR-T) therapy has achieved remarkable success in treating hematologic malignancies. The FDA approved three anti-CD19 CAR-T cell products: tisagenleucel (Kymriah), axicabtagen ciloleucel (Yescarta), and brexucabtagene autoleucel (Tecartus) for CD19+ B cell malignancies, such as B-ALL, diffuse large B cell lymphoma, and mantle cell lymphoma [76].

However, CAR-T therapies face significant challenges in solid tumors due to the hostile tumor microenvironment (TME), which features immunosuppression, physical barriers, metabolic constraints, and immune evasion mechanisms [77].

The TME contains immunosuppressive myeloid populations that inhibit CAR-T function. Myeloid-derived suppressor cells (MDSCs) accumulate within solid tumors and secrete immunosuppressive cytokines (TGF-β, IL-10), impairing T cell activation and cytotoxic function [78]. CAR-T infusion paradoxically promotes myeloid expansion through IFN-γ-mediated inflammation, creating a feedback loop that enhances immunosuppression and T cell exhaustion [78,79]. GM-CSF drives MDSC expansion and PD-L1 upregulation, causing CAR-T anergy [80], while reactive nitrogen species alter Lck function, hampering proliferation [81]. Similarly, tumor-associated macrophages (TAMs) polarize to an M2 phenotype, producing anti-inflammatory cytokines and expressing PD-L1 [82]. Increased TAM infiltration correlates negatively with remission rates, and their heterogeneous spatial distribution creates immunosuppressive niches throughout tumors [83].

Several strategies target myeloid-mediated suppression. Nab-paclitaxel depletes tumor-associated MDSCs and has been proposed as preconditioning chemotherapy [84,85]. Triggering receptor expressed on myeloid cells 2 (TREM2), upregulated in MDSCs and TAMs, activates SYK-PI3K/Akt/mTOR, MAPK, and NF-κB pathways, promoting immunosuppression. TREM2 blockade enhances anti-PD-L1 immunotherapy in murine models [86,87]. Chen et al. engineered CEA-specific CAR-T cells secreting bispecific scFvs against TREM2 and PD-L1, enabling localized intratumoral release that blocked both pathways, reduced MDSC and TAM proportions, and enhanced CAR-T proliferation [88].

Folate receptor beta (FRβ), widely expressed on M2-like TAMs, represents another promising target. Rodriguez-Garcia et al. engineered FRβ-targeting CAR-T cells that selectively lysed M2 TAMs in murine cancer models, increasing pro-inflammatory cytokines, CD8+ T cell infiltration, and delaying progression [89]. Notably, FRβ CAR-T preconditioning enhanced subsequent MSLN-CAR-T efficacy, suggesting that sequential targeting may potentiate solid tumor responses.

CD47 overexpression inhibits macrophage-mediated phagocytosis. While anti-CD47 antibodies restore tumor clearance, they also deplete CAR-T cells. Yamada-Hunter et al. engineered a CD47 variant (Q31P) that maintains SIRPα signaling but resists antibody binding, protecting CAR-T cells while enabling tumor targeting [90].

Solid tumor heterogeneity enables antigen escape through multiple mechanisms: selective outgrowth of antigen-negative clones under CAR-T pressure [82], genetic instability causing epitope loss, alternative splicing producing truncated proteins lacking CAR-binding domains, and deficits in antigen processing machinery (proteasome, TAP) reducing surface presentation [91]. Additionally, tumor dedifferentiation or transdifferentiation (e.g., epithelial-to-mesenchymal transition) can fundamentally alter antigenic repertoires, producing target-negative phenotypes [92].

T cell exhaustion results from impaired glycolytic capacity and mitochondrial dysfunction. The TME’s nutrient-depleted, hypoxic conditions force CAR-T cells toward less energy-efficient metabolic pathways. Exhausted cells upregulate multiple checkpoint receptors (PD-1, TIM-3, LAG-3, TIGIT), creating multidirectional functional inhibition resistant to single checkpoint blockades [93].

Cancer-associated fibroblasts (CAFs) produce abundant extracellular matrix components (collagen, glycosaminoglycans), creating dense fibrous networks that physically impede CAR-T cell infiltration. Despite effective diapedesis, stromal penetration remains inefficient [82].

Solid tumors exhibit irregular vessel architecture, abnormal endothelial junctions, and altered blood flow, impeding CAR-T cell extravasation [94]. These vascular abnormalities reduce oxygen delivery—exacerbating TME hypoxia and acidity—while limiting metabolic waste clearance (lactate, ammonia) [95].

**Figure 2:**
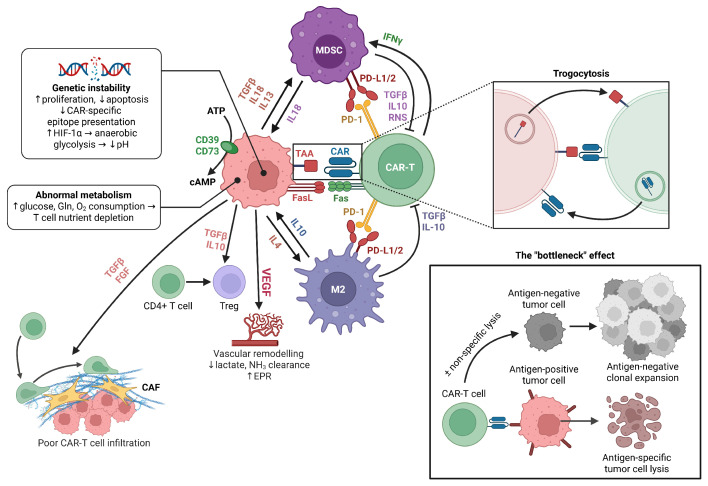
Schematic representation of the tumor microenvironment illustrating key cellular and molecular factors that limit CAR-T cell infiltration and antitumor activity. The figure highlights how genetic instability and abnormal metabolism in tumor cells drive nutrient depletion and immunosuppressive signaling, while stromal (CAF) and immune cell subsets (MDSCs, M2 macrophages, Tregs) secrete factors (TGFβ, IL-10, IL-4, VEGF) that suppress CAR-T cell function via checkpoint ligand expression (PD-L1/2, FasL), metabolic competition, and cytokine-mediated modulation. Mechanisms such as trogocytosis and the “bottleneck effect” are depicted as contributors to antigen escape and reduced CAR-T specificity, ultimately resulting in poor CAR-T cell infiltration, survival, and cytotoxicity. Created in BioRender. Tirziu, A. (2025), https://BioRender.com/513tk2a (accessed on 29 December 2025).

The multifactorial barriers and immune modulation in the tumor microenvironment affecting CAR-T cell efficacy are summarized in Figure 2.

Tumor-associated stressors (reactive oxygen species, hypoxia, acidosis) regulate exosome biogenesis, cargo composition, and paracrine signaling [96].

TME acidosis results from HIF-1α-driven anaerobic glycolysis and lactate accumulation. Despite extracellular acidity, intracellular pH (7.12–7.56) is maintained by V-ATPases, Na^+^/H^+^ exchangers, carbonic anhydrase IX, and bicarbonate transport [97,98,99]. V-ATPase disruption shifts MVB trafficking from lysosomal degradation toward exosome release, although this effect is cargo- and context-dependent [100]. Hypoxia upregulates HIF-1α and β, which triggers the transcription of Rab27a, a key protein involved in MVB trafficking [101]. Hypoxia also modulates the activity of sphingomyelinases, converting sphingomyelin to ceramide [102]. HIF-1α upregulates PKM2, which phosphorylates SNAP-23, a SNARE complex component that mediates the MVB–plasma membrane fusion and exosome release [103]. Additionally, STAT3 upregulation reduces Rab7 expression, while enhancing Rab27a production, favoring exosome synthesis and release [101].

The acidic, hypoxic TME impairs NK and T cell proliferation and cytotoxicity [104,105], limiting endogenous immune cell-derived exosome production. Direct exosome administration can bypass this limitation.

The small size of exosomes favors tissue penetration relative to cells, but physical extracellular matrix (ECM) barriers, abnormal tumor vasculature, and systemic clearance alter exosome biodistribution [82,106]. On the other hand, stromal density and ECM composition can impede nanoparticle/exosome diffusion and effective intratumoral penetration [107].

The enhanced permeability and retention (EPR) effect, along with leaky tumor vasculature, promotes the accumulation of nanoscale carriers in tumors. Exosomes naturally exploit these effects as nanocarriers. Quantitative in vivo tracking of administered exosomes labeled with ^89^Zr displayed predominant uptake in the liver and spleen, with only moderate distribution to other organs, so tumor-selective accumulation via passive EPR is not guaranteed [108]. Additionally, EPR is highly heterogeneous across tumor models and species. Exosome source, dose, and surface properties can influence tumor penetration or clearance [109].

Engineered immune cell-derived exosomes (dendritic cell, NK, T cell) carrying stimulatory molecules, antigens, or immunomodulatory cargo produce antitumor immune activation in preclinical studies. DC-derived platforms have entered early-phase trials with favorable safety and immunogenicity [110,111].

## 3. Exosomes

### 3.1. Exosome Biogenesis

Exosomes are 30–150 nm extracellular vesicles derived from multivesicular bodies (MVBs) via ESCRT-dependent and ESCRT-independent pathways. Unlike other extracellular vesicles that bud directly from the plasma membrane, exosomes undergo a unique biogenesis pathway involving two sequential membrane invagination events.

Early Endosome Maturation

Exosome biogenesis begins with the formation of early endosomes by plasmalemmal invagination during endocytosis. Early endosomes internalize molecules from the extracellular milieu that are processed and directed toward different cellular compartments. During their maturation phase, progressive acidification and late endosomal marker expression occur (Rab7/9, LAMP1, or CD63), transforming them into late sorting endosomes [112].

In the late sorting endosome phase, proteins, miRNAs, mRNAs, and other small molecules enter the endosomal lumen via various sorting mechanisms, determining the final composition of exosomes [113].

Multivesicular Body Development

The transformation of late endosomes into multivesicular bodies (MVBs) involves a second membrane invagination event that generates intraluminal vesicles (ILVs) within the endosomal lumen. This process requires specialized molecular machinery to drive inward (negative) membrane budding against the natural tendency for outward curvature [114].

MVBs represent the precursors of exosomes, with their ILVs serving as exosomal templates. The intraluminal content of MVBs resembles the extracellular milieu, while the ILV composition is similar to the cytosolic compartment (comprising miRNAs, mRNAs, and soluble proteins) [115].

#### 3.1.1. ESCRT-Dependent Biogenesis Pathways

The endosomal sorting complex required for transport (ESCRT) machinery represents the canonical pathway for ILV formation and cargo sorting. The ESCRT machinery consists of four main complexes (ESCRT-0, -I, -II, and -III) that function sequentially to recognize cargo, induce negative membrane curvature, and perform membrane scission.

The ESCRT-0 complex initiates the process by recognizing and clustering ubiquitinated cargo proteins at the endosomal membrane [116]. This Vps27/STAM heterodimer contains VHS domains (binding ubiquitinated proteins) and a shared FYVE domain (binding PI3P), enabling dual membrane–cargo attachment [117].

ESCRT-I and ESCRT-II complexes work cooperatively to deform the endosomal membrane and bridge cargo recognition with membrane remodeling. The ESCRT-I complex (TSG101/Vps28/Vps37/Mvb12) clusters ubiquitinated cargo and recruits ESCRT-II via Vps28–Vps36 GLUE domain interactions [116,118]. ESCRT-II facilitates membrane curvature generation and ESCRT-III recruitment by the Vps25–Vps20 interaction [119]. The ESCRT-III complex produces membrane scission through polymerization into helical filaments that constrict the membrane neck. Vps20 protein from the ESCRT-III complex interacts with the Vps25 ESCRT-II protein and functions as a primer for Snf7 polymerization. The polymerization process is stopped by Vps24, which caps the Snf7 terminal protein and recruits Vps2. Membrane fission completion is achieved by Vps4 AAA ATPase. Vps4 interacts with Vps2 and provides energy for ESCRT-III disassembly and membrane fission completion [120].

The ESCRT pathway uses multiple cargo recognition strategies to ensure selective protein sorting into ILVs. Ubiquitination serves as the primary sorting signal, with different ubiquitin linkages directing proteins toward degradation or exosomal secretion. Late domain motifs (P(S/T)AP, PPXY, YPX[n]L) found in the structure of cargo proteins provide direct binding sites for ESCRT adaptors, enabling ESCRT-independent recruitment of specific proteins [121]. ALIX acts as an alternative adaptor protein that binds to PPXY motifs. It connects cargo proteins to ESCRT-III components enabling the packaging of non-ubiquitinated proteins into exosomes, thereby expanding the exosome cargo repertoire [122].

##### The Syndecan–Syntenin–ALIX Pathway

Syndecan molecules present on the endosomal membrane interact with the protein syntenin via its two PDZ domains. The N-terminal segment of syntenin interacts with ALIX via the LYPX(n)L motifs in a 1:1:1 ratio (syndecan–syntenin–ALIX) [123]. ALIX joins the syndecan–syntenin complex with components of the ESCRT pathway, such as TSG101 (ESCRT-I) or CHMP4B (ESCRT-III), triggering endosomal membrane budding to become intraluminal vesicles [124].

#### 3.1.2. ESCRT-Independent Biogenesis Pathways

##### Ceramide Pathway

The ceramide pathway represents a major ESCRT-independent mechanism driven by lipid-induced membrane remodeling. Ceramide, a cone-shaped sphingolipid, induces negative membrane curvature when concentrated in membrane domains, promoting spontaneous ILV budding [114]. Neutral sphingomyelinase (nSMase) converts sphingomyelin to ceramide in early endosomes, while acidic sphingomyelinase (aSMase) catalyzes this reaction in late endosomes. Pharmacological aSMase inhibition significantly reduces ceramide production and exosome release. This mechanism preferentially sorts lipid raft-associated proteins and lipids, contributing to exosomes’ distinct lipid composition [125].

##### Phospholipase D2 and Phosphatidic Acid Signaling

PLD2, activated by ARF6 GTPase, hydrolyzes phosphatidylcholine to phosphatidic acid (PA). PA accumulation induces negative membrane curvature, particularly important in cells with high exosome secretion rates requiring rapid lipid turnover [126].

##### Tetraspanin-Enriched Microdomains

Tetraspanins (CD9, CD63, CD81, CD82, Tspan8) are four-pass transmembrane proteins generating specialized microdomains that regulate ESCRT-independent cargo sorting. These proteins form microdomains with other tetraspanins, transmembrane proteins, and specific lipids, undergoing coordinated internalization [127].

CD81’s cone-shaped structure contains an intramembrane cavity binding cholesterol molecules; tetraspanin–cholesterol complexes facilitate microdomain formation and inward budding [128]. CD63 exhibits dual functionality, triggering both ESCRT-independent and ESCRT-dependent sorting [129]. CD9 contributes to cargo selectivity by recruiting specific proteins (metalloproteinase CD10, β-catenin [130], integrin α2β1 [131]) and lipids into TEMs, creating distinct exosome subpopulations [132].

**Figure 3:**
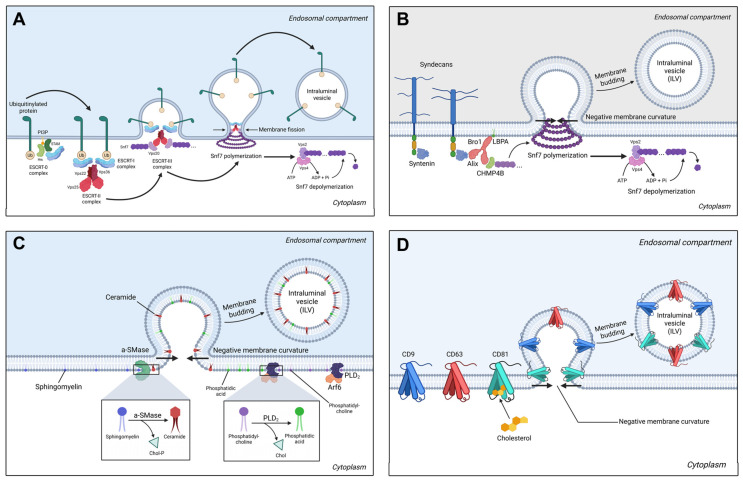
(**A**) The ESCRT-dependent exosome biogenesis starts with ubiquitinylated proteins that interact with the ESCRT-0 complex, comprising Vps27 (TSG101) and STAM. The ESCRT-0 complex is anchored to the endosomal membrane via the FYVE domains interacting with phosphatidyl-inositol-3-phosphate (PI3P). ESCRT-0 complex favors the interaction with the heterotetrameric ESCRT-I complex, which tethers the ESCRT-II complex. The ESCRT-II complex comprises the Vps36 protein that interacts with the ESCRT-I complex, and two Vps25 proteins. Vps25 binds the Vps20 protein of the ESCRT-III complex, serving as a primer for Snf7 polymerization. Upon its polymerization in a folded helix, the membrane presents a negative curvature that accentuates, leading to membrane budding and intraluminal vesicle formation. After vesicle release, Snf7 depolymerization occurs in the presence of Vps4, an AAA ATPase that generates the energy required for Snf7 depolymerization. Created in BioRender. Tirziu, A. (2025), https://BioRender.com/sylgtyo (accessed on 29 December 2025). (**B**) The syndecan–syntenin–ALIX pathway for exosomal biogenesis starts by the interaction of the intracytoplasmatic domains of syndecans with syntenin via the PDZ domains. Syntenin tethers ALIX, which interacts with TSG101, or with the CHMP4B protein of the ESCRT-III complex, leading to Snf7 polymerization and membrane budding. Created in BioRender. Tirziu, A. (2025), https://BioRender.com/1e4x1fz (accessed on 29 December 2025). (**C**) The ceramide pathway involves the hydrolysis of endosomal membrane sphingomyelin to ceramide by the acidic sphingomyelinase. The cone-shaped ceramide induces a negative membrane curvature, leading to membrane budding and ILV formation. The PLD2 pathway involves the action of phospholipase D2 activated by the Arf6 GTP-ase, which converts the endosomal phosphatidylcholine to phosphatidic acid, generating a negative membrane curvature. Created in BioRender. Tirziu, A. (2025), https://BioRender.com/vhxopci (accessed on 29 December 2025). (**D**) The tetraspanin pathway involves the transmembrane, cone-shaped proteins CD9, CD63, CD81 that generate a negative membrane curvature. Created in BioRender. Tirziu, A. (2025), https://BioRender.com/isbe9s1 (accessed on 29 December 2025).

The exosome biogenesis pathways are summarized in Figure 3.

#### 3.1.3. Cytoskeletal Transport Mechanisms

MVB intracellular transport requires cytoskeletal interactions involving actin filaments, microtubules, and molecular motors. MVBs undergo kinesin-mediated anterograde transport along microtubule plus ends toward the cell periphery and plasma membrane, essential for exosome secretion in polarized cells. Conversely, dynein-mediated retrograde transport moves MVBs along microtubule minus ends toward lysosomes for degradation. Dynein motors mediate this retrograde transport for the main degradation pathway of MVBs [133]. One remarkable regulatory mechanism of MVB trafficking toward lysosomes is ISGylation. ISGylation is the process of post-translationally linking interferon-stimulated gene 15 proteins (ubiquitin-like proteins) to molecules involved in MVB trafficking. ISGylation of TSG101 induces its aggregation and degradation, leading to reduced exosome secretion in T lymphocytes [134].

Cortical F-actin networks initially impede MVB membrane access, requiring localized depolymerization for docking and fusion. Actin-binding proteins (Arp2/3, HS1, Dia1, FMNL1) coordinate polarized secretory trafficking [135].

#### 3.1.4. Membrane Fusion Machinery

The final step in exosome secretion involves the fusion of MVBs with the plasma membrane, a process mediated by SNAREs (Soluble N-ethylmaleimide-sensitive factor Attachment protein REceptor), proteins that require calcium signaling.

SNARE Proteins, including SNAP23, VAMP7/8, YKT6, and various syntaxins, facilitate membrane fusion by forming trans-SNARE complexes.

Exosome secretion exhibits calcium dependence, with elevated cytosolic calcium levels promoting MVB–plasma membrane fusion. Hence, exosome release can be regulated by cellular signaling pathways involved in calcium homeostasis [114].

Rab GTPases function as molecular switches for exosome biogenesis and secretion, controlling vesicle budding, transport, and fusion through their ability to cycle between active (GTP-bound) and inactive (GDP-bound) states. Particularly, Rab27a and Rab27b are crucial for exosome secretion, as they localize to MVBs and recruit effector proteins that facilitate plasma membrane docking and fusion [136].

#### 3.1.5. Cargo Loading

The current literature provides evidence for an active selection process of the exosomal cargo. However, the detailed mechanisms of EV cargo selection are incompletely characterized.

Post-translational modifications are chemical changes in proteins occurring after translation, catalyzed by specific enzymes. PTMs covalently add functional groups that influence protein conformation, function, and subcellular localization. PTMs like phosphorylation, ubiquitylation, SUMOylation, N-glycosylation, palmitoylation, and ISGylation participate in the process of incorporating protein molecules into EVs. The post-translational modifications of proteins are summarized in Table 2.

Some proteins are ubiquitously found among exosomes, while other proteins are cell-specific. Common proteins include cellular adhesion molecules (CAMs), integrins, tetraspanins (CD63, CD9, CD81), MHC class I, Rab2/5/7, flotillin-1, annexins, heat shock proteins (Hsp70, Hsp90), cytosolic proteins involved in MVB formation (ALIX, TSG101), and cytosolic enzymes (GAPDH) [143,144]. Regarding immune cells, MHC class II molecules can be found on B lymphocytes and APC-derived exosomes [145], BCR and TCR are specific to B/T cell-derived exosomes, FasL and Apo2L can be found in T lymphocyte-derived exosomes, and perforin and granzyme A/B are associated with exosomes from CTL and NK cells [146,147].

The incorporation of microRNAs and mRNAs into exosomes involves specialized mechanisms that differ from those involved in protein sorting. Specific RNA-binding proteins (RBPs) facilitate the loading of specific RNA molecules into exosomes by recognizing structural motifs in target RNAs, known as EXO-motifs (GGAG, UGAG, CCCU, and UCCU) [148], while RAFT-motifs (CCCU, UCCC, CUCC, UUGU) [149] can directly interact with the lipid rafts enriched in ILV membranes.

### 3.2. Exosome Isolation Methods

Exosomes can be isolated from biological fluids (blood, urine, ascites, cerebrospinal fluid) or cell culture media. Beyond diagnostic biomarker applications, their therapeutic potential has opened new cancer treatment directions [150].

There are a variety of methods for isolating exosomes, each with its specific advantages and pitfalls in terms of yield and purity.

Ultracentrifugation (UC) is the reference method, using high centrifugal forces (≥100,000× *g*) to separate extracellular vesicles from cellular components. Despite high protein yields (~440 µg/mL), UC has limitations: it requires large sample volumes, risks protein/lipoprotein contamination, causes vesicle aggregation [151], and induces mechanical stress altering biophysical properties. Combining UC with size exclusion chromatography (SEC), affinity cleanup, density cushions, or lipoprotein removal beads (e.g., LipoMin) improves purity for proteomic or clinical applications [152,153].

Ultrafiltration separates vesicles by passing samples through porous membranes with defined molecular weight cut-offs under applied force [154]. Vesicles and molecules greater than the MW cut-off remain in the retentate, while the exosomes pass through the filtering membrane along with the solvent in the permeate. In dead-end ultrafiltration, centrifugal force drives small molecules and vesicles through the membrane in the force direction. One major limitation is the accumulation of filtered material on the membrane surface (filter cake), reducing the permeation rate, and membrane entrapment/adsorption, resulting in sample loss [150]. Hence, dead-end filtration is suitable for low sample volumes. In tangential flow filtration (TFF), the feed solution flows parallel to the membrane surface, while molecules and vesicles pass through a hollow fiber membrane perpendicular to this flow under hydrostatic pressure. As the feed solution flows parallel to the membrane surface, it prevents membrane clogging by vesicle aggregates [155].

Precipitation utilizes highly hydrophilic polymers, such as PEG (polyethylene glycol), to reduce exosomal solubility. PEG sequesters water molecules surrounding exosomes, creating a hydrophobic microenvironment that induces precipitation. EVs can be further separated from the aqueous solution with one centrifugation step (10,000× *g*). Precipitation methods were reported to concentrate exosome range particles approximately 2.5-fold more per mL and be six times faster than UC in a protocol comparison, with similar levels of lipoprotein contamination [156].

Affinity-based methods employ exosome-specific antibodies, providing high specificity and selectivity (with the possibility to isolate specific exosomal subpopulations [157]). However, they require large sample volumes and have the potential of losing certain EV subpopulations. Additionally, the use of non-neutral pH elution and isolation buffers to detach the antibodies might hamper their functionality [158].

Size exclusion chromatography (SEC) involves passing the sample through a chromatographic column filled with microbeads of a specific size. Smaller vesicles enter microbead pores and are delayed by vesicle–bead collisions, while larger particles bypass pores and elute faster. Time-based fraction collection enriches exosomal populations [159]. Since the sample passage occurs due to gravitational pull or low centrifugation speeds, the exosomal membrane’s integrity and functionality are maintained [157]. The large sample volume, lipoprotein contamination, and high costs for chromatographic columns are the main limitations of this method. The lipoprotein contamination issue can be addressed by combining SEC with lipoprotein removal agents (such as LipoMin), leading to an EV yield (measured by anti-CD81 ELISA) of approximately 93% [152].

In conclusion, there is no single universally best method. The choice depends on the experimental priority: yield, purity, speed, scalability, or specificity. Polymer precipitation or ultrafiltration approaches result in a much larger protein/RNA yield (5610 µg/mL protein vs. UC: 440 µg/mL), but with a lower purity due to more co-isolated contaminants [160,161]. Size exclusion chromatography (SEC) combined with density gradient centrifugation or affinity-based isolation results in a higher purity required for proteomics or clinical use [152,153]. Commercial precipitation kits or magnetic capture beads are simpler and faster approaches, but with a lower purity [152,162]. Finally, microfluidic or chip-based approaches carry the potential for clinical translation [151,160].

## 4. CAR-Exosomes

### 4.1. CAR-T Exosomes (CAR-T Exos)

CAR-T Exos may substitute CAR-T cells as cytotoxic agents, overcoming some CAR-T-related toxicities [10]. The application of CAR-T Exos in cancer therapy could make CAR therapies more clinically controllable and effective.

Since exosomes are cell-free, they are less likely to cause adverse reactions compared to CAR-T cells. In CAR-T manufacturing from leukemia patients, residual tumor cells may be inadvertently transduced with CAR. Using exosomes instead of whole CAR-T cells eliminates this risk, as exosome isolation removes all cellular contaminants including any CAR-transduced tumor cells [2,163].

CAR-T Exos simplify the anticancer strategy due to their nanoscale size. Exosomes can deliver therapeutic agents to sites where CAR-T cells cannot penetrate, especially in tumors with a significant fibrotic burden [164]. In cell-based approaches, cells need to actively migrate to tumor target sites. Exosomes, however, can be delivered through the circulation or other biological fluids, crossing biological barriers such as the blood–brain barrier and blood–tumor barrier. The ability of tumor-derived exosomes to traverse biological barriers and appear in circulation [165] suggests that exosomes possess intrinsic barrier-crossing properties. Studies with therapeutic exosomes confirm that they can cross the blood–brain barrier following systemic administration [166,167]. Furthermore, since exosomes are inherently present in the circulatory system and various tissues, their administration is considered safe, with minimal toxicological or inflammatory consequences [168].

CAR-T cells have shown less satisfying therapeutic effects in solid tumors compared to lymphoid malignancies. This difference reflects challenges in penetrating the tumor microenvironment and forming immune synapses [1].

Tumor killing requires CAR-mediated recognition of tumor antigens, followed by exosome–target cell interaction via membrane fusion or endocytosis. Upon internalization, cytotoxic cargo (perforin, granzymes, FasL) is released into the target cell cytoplasm, triggering caspase-dependent apoptosis [11]. CAR-exosomes exhibit many of the properties found in CAR-T cells, particularly in their killing effectiveness. Evidence supports that CAR protein levels are comparable between CAR-T Exos and their parental cells. In vitro cytotoxicity assays showed that 10 μg CAR-T Exos (containing 6 ng CAR protein) achieved equivalent tumor cell killing (20% killing of 5000 tumor cells) to 5 × 10^4^ CAR-T cells (10 ng CAR protein) [3]. CAR-T Exos possess a variety of cytotoxic molecules, including perforin, FasL, Apo2L, and granzyme A/B, while being negative for PD-1 expression. The absence of PD-1 enables them to resist the immunosuppressive influences of the tumor microenvironment better than their cellular counterparts. However, due to their non-replicative nature, multiple dosing regimens are required to achieve a significant tumor cell lysis. Since exosomes are cell-free, there is no potential for leukemic transformation and a lower risk for CRS (proven in murine models). According to Fu et al., the antitumor activity of CAR-exosomes is antigen-specific and mediated through CAR, as anti-EGFR and anti-Her2 CAR-exosomes effectively eliminate EGFR+ and Her2+ tumor cells [3]. Yang et al. developed MSLN-CAR-T Exos that suppressed tumor growth in vivo by the direct killing (perforin and granzyme B) of MSLN-positive TNBC without significant side effects [169].

Exosome pharmacokinetic properties are essential for therapeutic development, as these parameters determine dosing strategies and future clinical efficacy. Exosomes enter target cells through multiple mechanisms: (1) direct membrane fusion mediated by phosphatidylserine, sphingomyelin, tetraspanins, and integrins [170]; (2) phagocytosis by macrophages and dendritic cells via scavenger receptor-mediated recognition; (3) clathrin-mediated endocytosis in tumor cells, cardiomyocytes, macrophages, and neural cells [112]; (4) caveolin-mediated endocytosis in endothelial cells, smooth muscle cells, and fibroblasts; and (5) macropinocytosis driven by Rac1, cholesterol, and ion flux. Macrophage-mediated exosome clearance is similar to apopotic cell clearance—via negative charge recognition (phosphatidylserine) by scavenger receptor A (SR-A). SR-A blockade with dextran sulfate decreased liver clearance while increasing the exosomal accumulation in breast cancer tissue (murine model) [171].

Exosome distribution is influenced by size, charge, surface properties, and target receptor expression. Following intravenous administration, exosomes cross endothelial barriers, interact with blood cells, and accumulate predominantly in the liver, spleen, and lymph nodes. Non-human primate studies demonstrate that intravenously administered exosomes achieve CSF penetration within 30–60 min and hepatosplenic accumulation within one hour [168]. At higher doses (9 × 10^12^ particles), exosomes cross the blood–CSF barrier. Intrathecal administration yields a shorter half-life (12.5 min).

Exosomes undergo rapid clearance via macrophage-mediated endocytosis in the liver, spleen, and kidneys [172]. Plasma half-life varies by source—for B cell-derived exosomes it is ~2 min [173], while for platelet-derived exosomes it is ~5.5 h [174]. Despite rapid plasma clearance, exosomes persist in tissues (e.g., spleen) for up to two hours and are detected in PBMCs—particularly CD20^+^ B cells and granulocytes—within one minute of injection. Repeated administration accelerates clearance due to the adaptive immune responses and exosome-specific antibody formation. Strategies to evade macrophage uptake include CD47, CD31, or CD24 surface modification [175]. Notably, exosomes expressing CD47 similar to tumor cells avoid clearance by macrophages in the bloodstream, which extends their circulation time and leads to increased accumulation in the target tumor tissue [176].

Exosomes demonstrate minimal toxicity, with no significant increases in inflammatory cytokines (IL-6, TNF-α, IL-1β) reported [168,177]. Their natural presence in biological fluids contributes to this favorable safety profile.

Exosomes aggregate due to poor zeta potential. Storage at −80 °C is recommended, as pH, temperature, and freeze–thaw cycles affect integrity and uptake efficiency [178]. Protease inhibitors and vortexing improve recovery.

While these pharmacokinetic principles apply to exosomes generally, CAR-T exosome-specific parameters remain incompletely characterized. Whether CAR expression alters biodistribution, clearance kinetics, or immunogenicity requires investigation.

Limited data exist regarding CAR-T exosomal cargo. To understand potential CAR-T exosome cargo, data from wild-type T exosomes can be analyzed, as CAR-T Exos retain core T exosome characteristics. Several studies provide evidence for T cell exosomal cargo. Exosomes produced by T cells, particularly upon T cell receptor (TCR) activation, contain components of the TCR complex, adhesion molecules, tetraspanins, chemokine receptors, and signaling proteins.

Exosomes derived from activated human T cells, including peripheral blood T cells, T cell clones, and Jurkat T cells, contain key components of the TCR complex, such as the TCR β-chain, CD3ε, and the ζ-chain. The detection of the phosphorylated ζ-chain within these exosomes indicates parental cell activation [179]. Additionally, T cell-derived microvesicles express adhesion molecules, including CD2, LFA-1, and CD18. These exosomes also express major histocompatibility complex (MHC) class I molecules and, to a lesser extent, MHC class II molecules. Chemokine receptor CXCR4 is consistently present on T cell-derived exosomes [180]. Proteins associated with signal transduction, such as Src family tyrosine kinases (Fyn and Lck), have also been identified within these vesicles [181].

By analyzing the ExoCarta and VesiclePedia databases, 155 microRNAs were identified in T-derived exosomes, which can be classified into functional groups, including TLR signaling modulators, differentiation regulators, proliferation/survival controllers, metabolic reprogrammers, and antiviral/stress response mediators (Table 3).

Molecules like miR-155 and miR-146a are involved in modulating TLR signaling and NF-κB activation. When packaged in T cell exosomes, these miRNAs can be transferred to other immune cells to maintain immune homeostasis [182,183]. The let-7 and miR-181 family regulate T cell differentiation and activation. Let-7 miRNAs control the transition from naive to effector T cells, while miR-181 family members regulate TCR signaling sensitivity and CD4+ T cell subset specification. Exosomal transfer of these miRNAs can influence the differentiation fate of recipient T cells [184]. The miR-15/16 and miR-17-92 clusters influence T cell proliferation and survival. These miRNAs control key cell cycle checkpoints and apoptotic pathways, ensuring proper T cell expansion during immune responses. Their exosomal transfer can modulate the proliferative capacity and survival of recipient T cells [185,186].

During T cell activation, metabolic reprogramming is essential for supporting biosynthetic demands. Metabolic miRNAs like miR-33a/b and miR-122 control lipid metabolism, glucose utilization, and mitochondrial function in T cells. Exosomal delivery of metabolic miRNAs can coordinate metabolic states across T cell populations [187,188]. miR-150 and miR-155 are key regulators of antiviral immunity, while miR-34a mediates p53-dependent stress responses [189].

**Table 3:** MicroRNAs found in exosomes from T cells and their functional roles.

Functional Group	MicroRNAs	Role in Exosomal Cargo	Key Mechanisms	References
Immune Regulation and Suppression	miR-155, miR-146a, miR-146b-5p, miR-21, miR-21, miR-142-3p, miR-142-5p, miR-223, miR-223, miR-150, miR-150	Maintain immune homeostasis and prevent excessive inflammation; transferred to recipient cells to modulate immune responses	Target NF-κB pathway components (IRAK1, TRAF6), regulate TLR signaling, suppress pro-inflammatory cytokine production	[182,184,190]
T Cell Activation and Polarization	let-7a, let-7b, let-7c, let-7d, let-7f, let-7g, let-7i, let-7i-5p, miR-181a, miR-181a, miR-181b, miR-181c, miR-181d, miR-29a, miR-29b, miR-29c,	Control CD4+ T cell differentiation into Th1, Th2, Th17, and Treg subsets; regulate T cell activation thresholds	Target lineage-specific transcription factors (T-bet, GATA-3, RORγt, Foxp3), modulate TCR signaling sensitivity	[184,186,191]
Cell Cycle and Apoptosis Control	miR-15a, miR-15a, miR-15b, miR-16, miR-16-2, miR-17, miR-19a, miR-19b, miR-19b-1, miR-19b-3p, miR-20a, miR-20a, miR-20b, miR-18b, miR-34a, miR-34b	Regulate T cell proliferation, survival, and programmed cell death; control cell cycle progression in activated T cells	Target cell cycle regulators (Rb, E2F, cyclin D1), pro-apoptotic factors (PTEN, p53), and anti-apoptotic proteins (Bcl-2)	[185,186]
Metabolic Regulation	miR-33a, miR-33b, miR-122, miR-26a, miR-26b, miR-27a, miR-27b, miR-103, miR-107, miR-148a, miR-148a, miR-148b	Control cellular metabolism, lipid homeostasis, and energy production; regulate metabolic reprogramming during T cell activation	Target metabolic enzymes (SREBP, ACC1, FASN), glucose and lipid metabolism pathways, and mitochondrial biogenesis	[187,188]
Antiviral and Stress Response	miR-34a, miR-150, miR-150, miR-132, miR-132, miR-155, miR-125a-3p, miR-125b, miR-125b-1, miR-125b-2, miR-100, miR-101, hiv1-miR-H1	Mediate cellular responses to viral infections, oxidative stress, and environmental challenges; coordinate antiviral immunity	Target viral replication machinery, interferon signaling pathways, stress-response genes, and autophagy regulators	[183,189]
Angiogenesis and Tissue Repair	miR-126, miR-210, miR-200a, miR-200b, miR-200c, miR-23a, miR-23b, miR-24, miR-25, miR-25-3p	Regulate vascular development, tissue repair, and wound healing responses; coordinate T cell migration and tissue homing	Target angiogenic factors (VEGF, Ang-1), EMT regulators (ZEB1, ZEB2), and matrix metalloproteinases	[192,193,194]
Epigenetic Regulation	miR-22, miR-128, miR-134, miR-138-2, miR-153, miR-301a, miR-301b, miR-326, miR-340	Control chromatin remodeling, DNA methylation, and histone modifications; regulate epigenetic memory in T cells	Target DNA methyltransferases (DNMT), histone deacetylases (HDAC), and chromatin remodeling complexes	[195,196]

While these cargo components characterize wild-type T cell exosomes, CAR modification may alter exosomal composition. Preliminary studies suggest that CAR-T Exos retain core T cell molecules while expressing surface CARs and enhanced cytotoxic cargo [197]. Comprehensive characterization of CAR-T exosome cargo remains a priority for understanding their therapeutic mechanisms.

Apart from their intrinsic cytotoxicity, CAR-exosomes can be loaded with various therapeutic agents for targeted tumor delivery [6,198]. CRISPR-Cas9 loading allows knockout for genes involved in tumor cell proliferation and survival. This enables a combined therapeutic strategy where the intrinsic cytotoxicity of the exosome works synergistically with the anticancer effects of the delivered cargo [199]. Hydrophilic molecules, such as nucleic acids (miRNA, siRNA, mRNA), can be loaded in exosomes using physical methods (electroporation) or chemical disruption (lipofection), while hydrophobic molecules can be loaded by a short period of direct co-incubation [199,200]. Johnson et al. provided evidence that CAR-T Exos loaded with RN7SL1 (a DAMP that activates RIG-I/MDA5 signaling in immune cells) reduce the proportion of MDSCs and TGF-β levels, while promoting the activation and expansion of tumor-specific T cells and enhancing the immunostimulatory phenotype of dendritic cells [201]. CAR miR Jurkat cells were engineered to produce and deliver therapeutic miR-34a directly within the tumor environment to enhance the killing of glioblastoma cells. Isolated exosomes from CAR-J-miR-34a cells, enriched with miR-34a-5p, significantly reduced U87 cell viability by downregulating CDK6 and JAK2 expression, resulting in a significant 30% reduction in cell numbers [202]. Experimental results by Hu et al. showed that CAR-T Exos significantly reduced the viability of MDA-MB-231 and HCC827 cells in vitro, both in isolated tumor cells and solid tumor models. Furthermore, CAR-T Exos exert cytotoxic effects on hematological tumor models. Anti-CD20 CAR-T Exos were shown to induce apoptosis in Raji cells [203]. Zheng et al. engineered hybrid nanovesicles called LipCExo@PTX by fusing anti-mesothelin (MSLN)/anti-PD-L1 bispecific CAR-T Exos with lung-targeted liposomes loaded with paclitaxel. Exosomes expressed high lung tissue tropism and tumor cell specificity. Following intravenous injection, 95% of nanovesicles accumulated in the lung. Anti-MSLN CARs facilitated paclitaxel delivery and cytotoxic granules to tumor cells, while anti-PD-L1 CARs efficiently reversed the immunosuppressive effect of the tumor microenvironment. Inhaled paclitaxel-loaded CAR-T Exos efficiently distributed to the lung and inhibited tumor growth while avoiding systemic toxicity. CAR-T Exos increased the number of CD8+ T cells with subsequent elevated levels of TNF-α and IFN-γ in the tumor microenvironment [204].

The efficacy of CAR-T Exos can be further enhanced by optimizing the production methods. Similarly to TCR-induced exosome enrichment, higher levels of CAR-T Exos can be achieved through various antigen stimulation techniques [205]. (CAR)-T cell activation induces the release of EVs enriched in FasL and Apo2, molecules involved in caspase-dependent apoptosis [206].

Tumor antigen heterogeneity and escape mechanisms limit CAR-T efficacy. The SUPRA-CAR system addresses this by producing exosomes with exchangeable specificity from a single cell source [207]. SUPRA-CAR comprises a universal receptor (zipCAR) and modular antigen-binding domain (zipFv). Swapping zipFv components redirects exosomes to different tumor antigens, enabling personalized therapy.

In addition to direct tumor targeting, engineered exosomes serve as tools for CAR-T cell manufacturing and maintenance. For example, engineered exosomes can be used for ex vivo activation and expansion of CAR-T cells in an antigen-specific manner [208]. This method can lead to higher purity and faster production of CAR-T cells, preventing their terminal differentiation and exhaustion. CD-19-positive extracellular vesicles improved the persistence and activity of CAR-T cells by upregulating surface CAR molecules. This led to improved tumor clearance in murine models, with a significant increase in circulating IFN-γ, but without significant increases in IL-6 and TNF-α. CAR-T cells stimulated by CD19+ EVs expressed better proliferation and antitumor activity in vivo, without additional adverse effects. Furthermore, exosomes with CAR ligands significantly promote the durability of CAR-T cells and reduce disease relapse. While controlled CAR-T stimulation via CD19+ EVs enhances activity [209], excessive or chronic stimulation via persistent CAR ligand exposure risks exhaustion [210]. Optimal dosing and timing strategies must balance activation and exhaustion risk.

Exosomes can also be used for in situ CAR engineering. By co-expressing surface anti-CD3/CD28 scFv with the dual ability to activate T cells and deliver CAR mRNAs, exosomes can efficiently activate and transfect T cells [211].

IL2-tethered small EVs (IL2-sEVs) derived from engineered Jurkat T cells have been shown to enhance CD8+ T cell anticancer activity and reduce PD-L1 expression in melanoma cells, thereby reshaping the immunosuppressive tumor microenvironment [212]. This finding suggests that IL-2-loaded CAR-T Exos may enhance CD8+ T cell-mediated tumor killing by increasing IL-2 bioavailability in the tumor microenvironment while reducing systemic exposure.

Another important aspect is to analyze the phenotype of the parental cells. Wang et al. found that exosomes from exhausted CD8+ T cells can be internalized by normal CD8+ T cells, impairing their proliferation (measured by Ki67 expression) and effector function (CD69 expression). Consequently, low IFN-γ and IL-2 expression correlate with tumor progression [209].

Since parental cell phenotype critically affects exosome function, not all exosomes enhance antitumor immunity. CAR-T cells are a heterogeneous population consisting of CD8+ and CD4+ CAR-T cells, so the exosomal population is also heterogeneous. Although CAR-T CD4+ and CD8+ exosomal cargo remains uncharacterized, wild-type counterpart data suggest functional differences. CD4+ T exosomes enhance the antitumor response of CD8+ T cells without influencing the activity of Tregs via miR-25-3p, miR-155-5p, miR-215-5p, and miR-375 [213]. T helper cell exosomes are enriched in TCR, participating in the immunological synapses that involve B cells as antigen presenting cells through MHC II-dependent signaling [214]. CD45RO- CD8+ T exosomes released more miR-765 than their CD45RO+ counterparts, limiting the estrogen-driven development of endometrial cancer via the miR-765/ER-β/PLP2/Notch axis [215].

**Figure 4:**
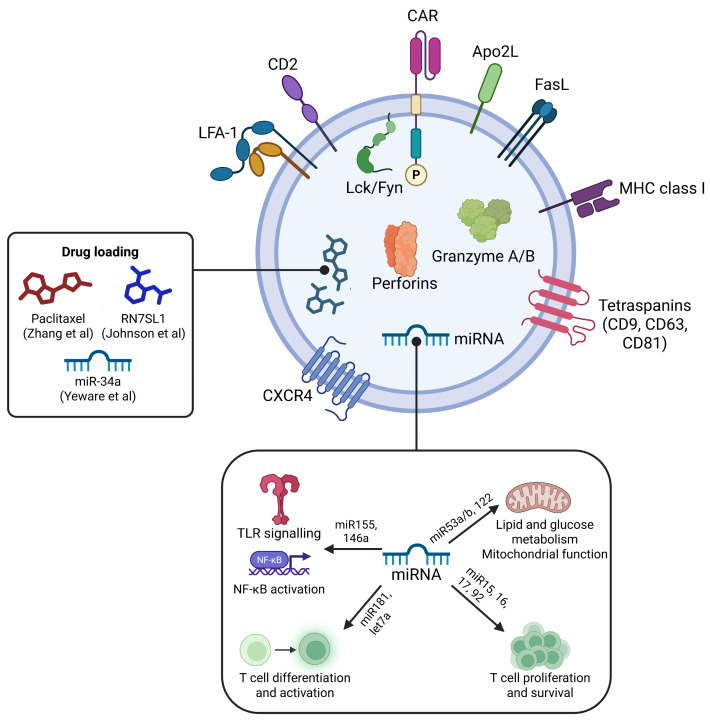
Molecular composition of CAR-T cell-derived exosomes. The exosomal surface displays the chimeric antigen receptor (CAR) responsible for tumor antigen recognition, along with key adhesion and signaling molecules including CD2, LFA-1 (lymphocyte function-associated antigen-1), and CXCR4 chemokine receptor, facilitating cellular interaction and migration. The exosome membrane also expresses the pro-apoptotic Apo2L and FasL ligands, major histocompatibility complex class I (MHC I) molecules for antigen presentation, and tetraspanins CD9, CD63, and CD81 for exosome biogenesis and cargo sorting. Internal cargo includes cytotoxic effector proteins granzyme A/B and perforin, which mediate target cell lysis. Associated signaling kinases Lck and Fyn phosphorylate downstream targets to promote activation. The exosomes carry microRNAs (miRNAs) such as miR-155 and miR-146a that activate TLR/NF-κB pathways, miR-181 and let-7a that regulate T cell differentiation and activation, and miR-15, miR-16, and miR-92 involved in lipid/glucose metabolism and mitochondrial function, contributing to T cell proliferation and survival. Therapeutic drug loading cargo such as paclitaxel, RN7SL1, and miR-34a highlight the potential of CAR-T Exos as delivery vehicles. Created in BioRender. Tirziu, A. (2025), https://BioRender.com/n137ly7 (accessed on 29 December 2025) [84,201,202].

The molecular cargo of (CAR)-T exosomes is described in Figure 4.

#### Limitations of the Literature and Future Directions

Despite promising preclinical data, CAR-exosome clinical translation faces substantial challenges spanning manufacturing, biodistribution, tumor microenvironment interactions, and regulatory pathways.

Clinical-scale exosome production requires 10^9^–10^10^ exosomes per patient dose [216,217], so upscaling requires large-scale bioreactor systems (hollow fiber bioreactors and stirred-tank bioreactors) for large cell cultures, extended culture periods for exosome accumulation, and efficient isolation methods capable of processing liters of conditioned media [218,219].

Variability in isolation, purification, and characterization methods of exosomes leads to inconsistent data and challenges in reproducibility, undermining the methodological rigor and comparability across studies [1,4,11]. Standardization is critical to ensure comparability of results, reproducibility, and reliable interpretation of exosome biogenesis and functional cargo, which currently limits translational progress [4,9].

The exosome cargo composition (proteins, miRNAs, lipids) is highly influenced by parent cell activation state, cell culture conditions (serum content, culture media composition), isolation methods, storage conditions, and duration [3,220,221]. These variables introduce complexity in the process of exosome production. Unlike CAR-T cells, where CAR expression and T cell phenotype define the product, CAR-exosomes require multi-parameter characterization (CAR density, cytotoxic protein content, size distribution, purity) with limited standardization across laboratories [150].

While potentially cheaper than autologous CAR-T production (no patient-specific manufacturing), allogeneic CAR-exosome production still requires GMP-grade CAR-T cell master banks, expensive isolation, storage (ideally, at −80 °C [222]), and transport equipment. Increasing exosome yield can be achieved by using immortalized producer cell lines (e.g., HEK293T, NK92, Jurkat cells) for continuous exosome production, reducing batch variability, and enabling large-scale manufacturing. A solution for the storage and transport limitations is to develop lyophilized CAR-exosome formulations for room-temperature storage, eliminating the need for storage and transport at low temperatures [223].

As already discussed, hepatosplenic clearance limits tumor accumulation, so several strategies to improve tumor penetrability or to reduce macrophage-mediated clearance can potentially improve exosome pharmacokinetics. Coating exosomes with PEG reduces macrophage uptake but may also impair target cell interaction [224]. Additionally, Yamada-Hunter et al. demonstrated that CD47-engineered T cells (and their exosomes) evade phagocytic clearance, improving biodistribution [90]. Furthermore, the incorporation of tumor-homing peptides (iRGD for integrin-expressing tumors [225,226]) or the use of bispecific CARs enhance tumor biodistribution. Alternative administration routes that bypass hepatosplenic clearance may be considered. Intratumoral injection achieves high local concentrations but is limited to accessible tumors. Intraperitoneal delivery may be considered for peritoneal carcinomatosis, bypassing systemic clearance [227]. In case of CNS malignancies, intranasal administration enhances brain penetrability [168].

Another aspect that needs to be clarified is which patients will benefit most. The most likely patients that would benefit from this therapy include high-risk patients ineligible for CAR-T cells (elderly, comorbidities that exacerbate CRS), as a maintenance or adjuvant therapy post-CAR-T/chemotherapy, or in combination with checkpoint inhibitors.

### 4.2. CAR-NK Exosomes (CAR-NK Exos)

CAR-NK Exos are NK cell-derived extracellular vesicles that inherit cytotoxic proteins and engineered targeting from parental CAR-NK cells, offering a cell-free, potentially safer alternative to parental CAR-NK cells and CAR-T cell therapies [228]. CAR-NK Exos can overcome adverse reactions like cytokine storm induced by CAR-T cell immunotherapy, while their enhanced tissue-infiltrating ability gives them an advantage in treating solid tumors. They combine NK cell cytotoxicity with targeted recognition by CAR molecules [229,230]. They are also easier to store and transport [203]. Additionally, CAR-NK exosomes do not express TCR molecules, therefore minimizing the risk of graft-versus-host disease even more. Another advantage of CAR-NK Exos is their recognition capability via non-specific receptors, such as NKG2D and NKG2A, which allow tumor cytotoxicity even in the presence of antigen downregulation [231]. CAR-NK Exos are an emerging area in cancer immunotherapy, and several studies have demonstrated their activity in in vitro and in vivo murine models, with additional insights extrapolated from wild-type NK-derived exosomes. Most cargo characterization derives from wild-type NK exosomes, as CAR-NK exosome-specific data remain limited. Whether CAR engineering alters exosomal composition beyond surface CAR expression requires investigation.

NK-derived exosomes (NK Exos) carry a complex cargo of cytotoxic proteins (perforin, granzyme B, FasL, TRAIL), surface receptors (NKG2D), RNAs, and lipids that mediate direct tumor killing and immune modulation. Proteomic and targeted assays identified that these proteins correlate with in vitro and in vivo cytolytic function [232,233]. EV membranes also display tetraspanins (CD63/CD81), adhesion molecules, and NK surface receptors that influence biodistribution and target cell interactions [234,235]. Preclinical models report enhanced tumor uptake, induction of apoptosis pathways, and modulation of signaling cascades. However, scalability, stability, and targeting specificity remain key hurdles [167,228].

NK Exos induce apoptosis in tumor cells via perforin/granzyme A/B activity, activating both caspase-dependent and -independent pathways [233,236]. They inhibit pro-survival kinases such as AKT and ERK and activate pro-apoptotic proteins including caspase-3/7/8/9 and PARP, with support shown in hepatocellular carcinoma models [232].

NK Exos contain a diverse array of cytokines, including TNF-α, IL-10, IFN-γ, chemokines (CCL3, CCL4, CCL5, CXCL1), and growth factors such as GM-CSF. These cytokines mediate interactions with macrophages and dendritic cells, thus playing a crucial role in immune modulation and response [167,230]. NK exosomes also stimulate T cell proliferation, and promote dendritic cell maturation, which subsequently activates NK cells via IL-12 [237].

NK Exos are characterized by the presence of FasL, capable of inducing apoptosis in target cells. Additionally, the activating receptor NKG2D recognizes stress-induced ligands expressed on tumor cells such as MICA/B and ULBPs. Importantly, NK-derived exosomes retain functional integrity despite TGF-β exposure. In contrast, TGF-β downregulates NKG2D in parental NK cells, weakening immune surveillance [235].

MicroRNAs carried by NK Exos contribute significantly to their function. For example, miR-186 exhibits tumor suppressor properties and correlates with NK cell activation markers such as NKG2D and DNAM-1 [238]. Other microRNAs—miR-146a-5p, miR-10b-5p, miR-92a-3p, and miR-99a-5p—attenuate tumor radioresistance by targeting signaling pathways such as ATM/ATR, PKB/mTOR, and JAK [239]. DiPace et al. identified a high expression of mRNA let-7b-5p in NK exosomal cargo, targeting cyclin-dependent kinase CDK6 and suppressing pancreatic cancer cell proliferation [240].

Exosomes from activated NK cells (primary or NK92), stimulated with IL-2, IL-12, and IL-15, display increased penetrability, solid tumor targeting capacity, and enhanced cytotoxicity against tumor cells, attributed to granzyme B and H enrichment [241,242].

CAR-NK Exo pharmacokinetics is critical for therapeutic optimization, though CAR-NK-specific data are scarce. Wild-type NK exosomes exhibit rapid systemic clearance (plasma t_1/2_ < 2 min in mice) following intravenous administration, with >90% accumulating in the liver and spleen within minutes via macrophage-mediated uptake. Transient binding to platelets and erythrocytes (~5 min) precedes hepatosplenic sequestration by Kupffer cells and splenic macrophages. Systemic inflammation dramatically extends their circulation (plasma t_1/2_ > 600-fold increase in LPS-primed mice) and enhances tissue penetration [243]. These findings might suggest favorable outcomes in combination therapies with immune checkpoint inhibitors.

Surface engineering strategies modulate these properties—albumin-binding domain display extends circulation by reducing blood cell interactions [243], while CAR expression enhances tumor targeting specificity [167,244]. CAR expression on parental NK cells or surface modifications, such as inserting a transferrin-receptor peptide, can enhance tumor targeting and blood–brain barrier crossing. For example, ExoCAR/T7@Micelle used anti-Her2 CAR-NK Exos combined with a T7 peptide, which binds the transferrin receptor to target cerebral vascular endothelial cells. The exosomes were also loaded with an ROS-responsive nanobomb (mPEG-TK-Ce6@RSL3) that activates in tumors with high ROS levels. The ROS and RSL3 trigger ferroptosis, producing cytotoxic effects on Her2+ tumor cells. However, intrinsic CAR-NK exosome cytotoxic activity was not assessed in this study [167].

Exogenous modifications, such as drug loading with sorafenib [243] or cisplatin, also augment NK exosome cytotoxicity. Cisplatin-loaded NK exosomes demonstrate the targeted killing of SKOV3 ovarian cancer cells with reduced off-target organ damage compared to systemic drug administration [245]. Similarly, combining NK Exos with paclitaxel inhibits tumor cell proliferation while minimizing normal cell damage [246].

Encapsulation of doxorubicin (DOX) within exosomes may significantly diminish systemic side effects while blocking topoisomerase II activity specifically in target tissues. This strategy reduces nonspecific toxicity to highly proliferative normal cells (found in bone marrow, hair follicles, gastrointestinal tract) and cardiomyocytes, potentially preventing acute cardiotoxicity [247]. Exosome lipid bilayers facilitate targeted membrane fusion and internalization via adhesion molecules such as proteoglycans, integrins, and lectins. Additionally, DOX-loaded NK Exos exhibit anti-angiogenic effects by downregulating VEGF-A and increasing p53 gene expression, enhancing therapeutic efficacy [248].

NK Exos loaded with miR-30c have been shown to enhance NK cell cytotoxicity against lung cancer in vivo by promoting IFN-γ and TNF-α secretion [249].

NK Exos can also undergo chemical modifications. Nguyen Cao et al. encapsulated NaHCO3 and paclitaxel into exosomes. Upon tumor uptake, NaHCO_3_ generated CO_2_, facilitating drug release in breast cancer cells [250]. Wang et al. developed biomimetic core–shell nanoparticles with a dendrimer core loaded with therapeutic miRNA and a hydrophilic NK Exo shell. These nanoparticles delivered let-7a miRNA to neuroblastoma cells, inhibiting tumor growth [251].

While cytokine stimulation (IL-2, IL-15, IL-21) enhances exosome release, CAR stimulation effects on exosome production remain unreported. Primary NK cell lines have limited proliferative capacity, impacting exosome yield. Alternative platforms like NK92 cells can mitigate this limitation, although an extensive evaluation of exosomal cargo for potential pro-tumorigenic nucleic acids or proteins is needed [6].

Endogenous modifications further enrich exosomal cargo. Lentiviral transduction of NK92MI cells to express Bcl-2 siRNAs enhances the pro-apoptotic activity of EVs in breast cancer cells [252].

Several preclinical studies suggest that the systemic delivery of NK Exos or engineered exosome constructs inhibits tumor growth in orthotopic and metastatic models [167,234]. As the perforin/granzyme pathway mediates CAR-NK cell cytotoxicity, preserving these effectors in exosomal cargo is critical for antitumor potency.

Although there are no current clinical trials on CAR-NK exosomes, preclinical results show promising applicability. Preclinical models have shown targeted delivery and effective tumor control, even in difficult sites like Her2+ brain metastases, highlighting the potential benefits in tissue penetration and safety [167]. Reviews comparing CAR-NK and CAR-T therapies highlight the favorable safety profile of CAR-NK, suggesting that exosome derivatives may further reduce cell-associated risks while enabling off-the-shelf use [228]. CAR-NK cells offer potent, sustained cytotoxicity and, in some designs, transient in vivo expansion, whereas exosomes provide dosing flexibility and enhanced tissue penetration [167,235]. As non-replicating entities, exosomes may lower the risks of cytokine storms or graft-versus-host disease compared to live cell products, though immunogenicity from engineered surface antigens and potential off-target effects require evaluation [230]. A single study evaluated the toxicity of CAR-NK Exos. Tao et al. examined the organs (heart, liver, spleen, lungs, and kidneys) of mice with breast cancer treated with CAR-NK exosomes loaded with ferroptosis inducers, reporting negligible toxicity following treatment [167]. A comparison of NK and CAR-NK exosomes is described in Table 4.

Exosome production shifts manufacturing challenges from cell expansion to high-throughput isolation and purification, both necessitating GMP pipelines. However, exosomes may simplify logistics related to storage and shelf life if stability is established [253,254]. Exosomes show particular promise for treating solid tumors and CNS metastases, or as carriers combined with nanoparticle cargos, where tissue penetration and minimal systemic toxicity are critical, as demonstrated in engineered CAR-NK Exo constructs for Her2+ brain metastases [167].

**Figure 5:**
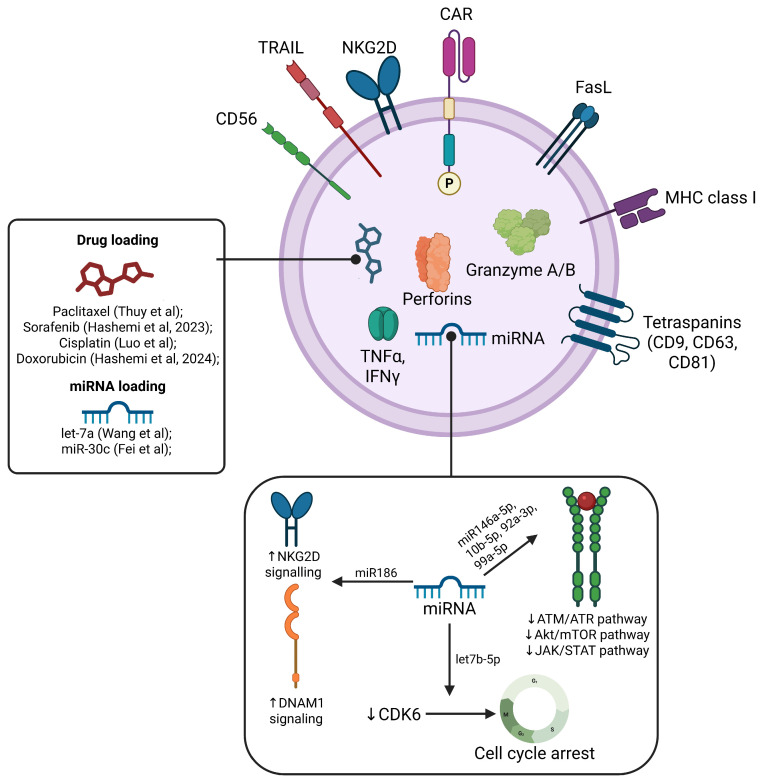
Surface molecules and cargo composition of CAR-NK cell-derived exosomes. The exosomal surface contains the chimeric antigen receptor (CAR) involved in tumor antigen recognition, along with activating receptors such as NKG2D and CD56, and pro-apoptotic ligands, TNF-related apoptosis-inducing ligand (TRAIL), and Fas ligand (FasL), which contribute to immune effector functions. Major histocompatibility complex class I (MHC I) and tetraspanins (CD9, CD63, CD81) are also expressed, common to exosome membranes and important for antigen presentation and cargo sorting. The internal cargo includes cytotoxic proteins granzyme A/B and perforins alongside immune cytokines TNF-α and IFN-γ, mediators of inflammation and tumor cell killing. The exosomes carry microRNAs (miRNAs) such as let-7b-5p, miR-146a-5p, miR-10b-5p, miR-92a-3p, and miR-99a-5p, which regulate multiple signaling pathways by downregulating ATM/ATR, Akt/mTOR, and JAK/STAT pathways, leading to cell cycle arrest via CDK6 inhibition. Additionally, miR-186 enhances NKG2D and DNAM1 signaling, boosting NK activation. Therapeutic cargo loading of chemotherapeutics was previously explored, using paclitaxel, sorafenib, cisplatin, and doxorubicin, alongside miRNAs let-7a and miR-30 [229,246,248,249,250,251].

The molecular cargo of (CAR)-NK exosomes is described in Figure 5.

**Table 4:** Comparison between NK and CAR-NK exosomes.

Feature	NK Exosomes	CAR-NK Exosomes
Primary functional cargo	Perforin, granzymes, death ligands, ncRNAs [6,232]	Same cytotoxic cargo plus engineered targeting moieties on surface when derived from CAR cells or modified [167]
Targeting specificity	Moderate tumor tropism via surface receptors [234]	Increased specificity if CARs or targeting peptides present on exosomes [167]
Therapeutic potency	Demonstrated in vitro/vivo tumor killing; potency limited by dose and distribution [234]	Enhanced in engineered platforms (e.g., HER2+ brain metastasis model) showing improved delivery and efficacy [167]
Safety profile	Lower theoretical systemic toxicity; non-replicating vesicles reduce cell-related risks [230,232]	Similar safety advantages; biodistribution and off-target binding still need assessment [167]
Manufacturing complexity	Easier storage/handling than cells but requires scalable EV isolation methods [254]	Additional engineering steps and stringent purification to ensure CAR display on EVs [167]

Despite promising preclinical results, several challenges must be addressed for clinical translation. The CAR-NK exosome-specific pharmacokinetic parameters, optimal dosing regimens, and comparative efficacy versus CAR-T Exos require systematic investigation. GMP-compliant manufacturing protocols for scalable production and standardized purification remain underdeveloped. The long-term stability, immunogenicity from repeated dosing, and potential off-target effects require comprehensive evaluation [253,254]. Early-phase clinical trials are needed to establish safety profiles, biodistribution in humans, and preliminary efficacy across tumor types. Addressing these gaps will accelerate clinical translation.

### 4.3. (CAR) Macrophage-Derived Extracellular Vesicles

CAR macrophage (CAR-M) therapy harnesses the remarkable plasticity of macrophages to target solid tumors. Macrophage-derived exosomes provide a promising cell-free platform for delivery and immunomodulation.

Macrophages exhibit distinct polarization states—M1 (pro-inflammatory) and M2 (immunoregulatory/pro-tumoral)—which critically influence the tumorigenic process and intercellular communication through Exos and other EVs. Genetically engineered CAR macrophages recognize tumor antigens, enhance phagocytosis, and remodel the TME, representing a therapeutic strategy distinct from CAR-T cell approaches [255].

Macrophage polarization profoundly impacts the composition and function of their derived exosomes. Exosomes isolated from M1 macrophages carry pro-inflammatory miRNAs and proteins, promoting immune activation and antitumor effects, whereas M2 macrophage-derived exosomes often contain immunosuppressive RNAs, including lncRNAs, that facilitate tumor progression and immune escape [256,257,258]. Therefore, isolating exosomes from M1-polarized macrophages is desirable for therapeutic applications.

At the molecular level, macrophage-derived EVs encapsulate diverse biomolecules such as cytokines, membrane receptors, microRNAs (miRNAs), lncRNAs, and messenger RNAs (mRNAs) that reflect their parental polarization state and modulate recipient cell functions [258,259]. MicroRNAs predominate among small regulatory RNAs and are differentially packaged based on the macrophage phenotype [258,260]. Additionally, M1 and M2 EVs carry distinct populations of other small non-coding RNAs, including isomiRs, tRNA fragments, piRNAs, sn/snoRNA fragments, and Y-RNA fragments 1. Functional lncRNAs and circular RNAs within these vesicles can further alter gene expression and cellular phenotypes in target cells [260,261].

Macrophage EV nucleic acids act as potent intercellular signals, influencing inflammation, metabolism, and cellular differentiation through defined molecular pathways. For example, M2-derived exosomal miR-216a-5p promotes M2 polarization by targeting TLR4 and suppressing the TLR4/NF-κB axis while activating PI3K/AKT signaling [262]. Conversely, M1 EVs enriched with miR-21-3p induce M1 polarization by regulating PTEN/PI3K/AKT pathways in alveolar macrophages [263]. Tumor-associated macrophage EV lncRNAs, such as HISLA, stabilize HIF-1α in cancer cells to enhance glycolysis and chemoresistance via the disruption of PHD2–HIF-1α interactions [261]. Furthermore, tumor-derived exosomal lncRNA TUC339 modulates macrophage activation and the expression of M1/M2 markers, underscoring bidirectional communication via lncRNAs [264].

To enhance tumor targeting, engineered macrophage EVs conjugated with dibenzocyclooctyne-modified antibodies targeting CD47 and SIRPα via pH-sensitive linkers accumulate selectively within the acidic tumor microenvironment, facilitating tumor cell recognition [265]. Moreover, M1 macrophage-derived exosomes (M1-Exos) delivering anti-PD-L1 siRNA effectively reduce PD-L1 expression in tumors, increase CD8+ T cell infiltration, and promote macrophage repolarization from the immunosuppressive M2 to the pro-inflammatory M1 phenotype, inhibiting tumor growth [266]. Additional strategies exploit EVs engineered to express OX40 ligand (OX40L), converting M2 macrophages to M1, and enhancing macrophage-mediated immunity in pancreatic adenocarcinoma models [267].

Macrophage-derived EVs can also deliver glycyl-tRNA synthetase (GARS1), which suppresses tumor growth by promoting M1 polarization and enhancing phagocytosis. GARS1-containing exosomes disrupt tumor cell adhesion through interactions with cadherin-6, inducing apoptosis [268]. Engineered EVs conjugated with mannose residues target M2 tumor-associated macrophages (TAMs) via CD206. When these EVs are loaded with drugs such as metformin, they can activate the AMPK-NFκB pathway to mediate repolarization to the M1 phenotype. Consequently, the antitumor response is augmented, particularly in combination with PD-1 inhibitors [269].

The delivery of ferroptosis-inducing agents like RSL3 via M1 EVs disrupts the redox balance by increasing oxidative stress and promoting tumor cell ferroptosis [270]. Macrophage-derived exosomes loaded with chemotherapeutics such as PTX improve drug efficacy and delivery against resistant cancer cells. Autologous exoPTX demonstrated enhanced potency in lung carcinoma metastasis models [270]. Modifications like conjugating aminotheylanisamide-polyethylene glycol (AA-PEG) improve drug loading and allow targeted accumulation in lung cancer cells, suppressing metastases and improving survival in murine models [271]. M1 macrophage-derived exosomes also exhibit superior drug loading efficiency and anticancer activity against both drug-sensitive and -resistant ovarian cancer cells compared to their M2 counterparts [272].

Functionally, M1 EVs promote tumor apoptosis and modulate the immunosuppressive tumor microenvironment. The mechanism involves the activation of caspase-3 and caspase-7, and the reduction in immunoregulatory markers such as CCR4, Foxp3, and CTLA-4 in immune cells [273]. In gastric cancer, M1 small EVs (sEVs) suppress tumor growth by activating T cells and downregulating PD-L1 expression. This process is mediated predominantly by miRNA miR-16-5p, which also enhances CD3+ and IFN-γ+ T cell populations [274].

In contrast, M2 TAMs foster tumor progression and immune evasion. Thus, therapeutic strategies aiming to repolarize M2 TAMs into M1 macrophages are critical for effective cancer treatment [275]. The intravenous administration of M1 sEVs in tumor-bearing mice successfully suppresses tumor growth by converting M2 to M1 macrophages within the tumor. This effect is markedly increased by enriching EVs with polarization-inducing miRNAs such as miR-155, miR-125, and miR-21 [275,276]. Furthermore, engineered M1-like macrophage exosomes expressing OX40L synergistically activate innate and adaptive immunity to inhibit tumor growth and metastasis in breast cancer mouse models [277].

Exosomes from M1 macrophages or engineered vesicles loaded with siRNA targeting PD-L1 have demonstrated the capacity to repolarize TAMs and restore CD8+ T cell activity in preclinical models [256,278].

CAR macrophages, the source of CAR-M exosomes, are recognized for their tumor microenvironment remodeling capabilities [279]. They exhibit a preference for an M1-like, pro-inflammatory, tumor-suppressive phenotype and stimulate the proliferation and activation of CD8+ cytotoxic T lymphocytes [280,281], suggesting that exosomes from these cells may inherit similar immunomodulatory properties.

Highlighting this potential, Jiang et al. developed CAR-M exosomes with increased CXCL10 expression, enhancing T lymphocyte activation and migration while promoting macrophage differentiation to the M1 phenotype. These exosomes were covalently conjugated with SN38, a drug that potentiates tumor cell cytotoxicity. This study provides evidence for a combined chemo-immunotherapeutic strategy using extracellular vesicles [282].

Overall, macrophage-derived EVs possess robust potential to modulate the TME through delivery of enzymes, cytokines, and regulatory nucleic acids that can either reprogram TAMs toward tumor suppression or, depending on the cargo, inadvertently promote tumor progression [255,256]. While (CAR-)M cell therapies may carry lower systemic cytokine release syndrome (CRS) risks, they exhibit limited persistence and potential liver sequestration, so exosome platforms offer promising alternatives with reduced cell-related risks, although rigorous cargo safety assessments are imperative [255].

### 4.4. CAR-Exosomes vs. CAR Cells

CAR-Exos have demonstrated antitumor activity in preclinical models. However, the critical assessment of their comparative efficacy, durability, and mechanistic equivalence to parent cells remains essential for realistic clinical translation.

CAR-T cells continuously produce IFN-γ, TNF-α, and other effector cytokines upon antigen encounter, amplifying local inflammation and recruiting innate immune cells. CAR-Exos deliver a single cargo load without adaptive response capacity. Each CAR-T cell can engage multiple tumor cells, while CAR Exos provide single-dose cargo delivery, requiring higher exosome–tumor cell ratios to achieve comparable effects. Exosomal cytotoxic protein content (perforin, granzymes, FasL) varies with parent cell activation state and culture conditions, introducing batch-to-batch variability.

The most significant limitation of CAR-exosomes is their inability to expand in vivo—a property that both defines CAR-T cell therapy’s success and increases the risk for toxicity. Living CAR-T cells proliferate extensively upon antigen encounter, establishing long-term immune surveillance that can persist for months [283]. In contrast, CAR-exosomes provide a non-renewable, single-dose effect. Pharmacokinetic data reveal rapid clearance: radiolabeled exosomes show plasma half-lives of 2–6 h in mice, with 70–85% cleared within 24 h [108].

The clinical implications are significant regarding tumor recurrence, patient burden, and the effect on minimal residual disease (MRD). Since CAR-Exos are non-replicative, tumors may relapse between exosome doses. In the case of MRD, CAR-Exos’ transient activity may be insufficient for MRD eradication. Frequent dosing increases treatment burden, healthcare costs, and potential for cumulative toxicities. One potential solution is a combination strategy where CAR-exosomes provide initial tumor debulking followed by CAR-T cell consolidation.

CAR-Exos represent a “snapshot” of parent cell functionality. Surface CAR expression enables antigen-specific targeting, cytotoxic proteins (perforins, granzymes, FasL) induce cell death, while miRNAs potentially reshape the tumor microenvironment. Due to their transient nature, CAR-Exos are unable to sustain cytokine secretion and paracrine signaling. Additionally, their cargo composition is not altered by antigen density or TME conditions.

These differences suggest that CAR-exosomes cannot replicate the full spectrum of CAR-T cell functions. They may be most effective in contexts where transient, targeted cytotoxicity is sufficient (e.g., adjuvant therapy post-surgery, combination with checkpoint inhibitors) rather than as monotherapy for well-developed tumors.

Suboptimal tumor accumulation represents a major translational obstacle. Biodistribution studies using radiolabeled exosomes (^89^Zr) reveal that 40–60% of injected exosomes accumulate in the liver and spleen within 2–6 h, mediated by Kupffer cell and splenic macrophage uptake [108,168]. This contrasts with CAR-T cells, which actively migrate to lymphoid organs and tumor sites via chemokine gradients (CCR7, CXCR3, CCR4) and can expand locally upon antigen encounter. CAR-exosomes rely primarily on passive EPR-mediated accumulation and CAR-mediated retention, which is insufficient in many solid tumors with poor vascularization or dense stroma.

Possible strategies to improve exosome biodistribution include surface modification with PEG or CD47 to reduce macrophage uptake [265], incorporation of tumor-homing peptides (iRGD) [225], magnetic guidance using iron oxide nanoparticles [284], or locoregional administration (intratumoral, intraperitoneal) to bypass systemic clearance [285]. These approaches remain in early preclinical development.

While CAR-exosomes lack replicative capacity, their safety profile is not without concerns. Exosomal cargo (cytotoxic proteins, inflammatory miRNAs) may affect non-target cells if exosomes are uptaken non-specifically. Similarly to CAR-T cells, these exosomes may bind target antigens also expressed on normal tissues, thereby delivering cytotoxic cargo to healthy cells. Consequently, exosomal miRNAs can alter gene expression in recipient cells, with unpredictable consequences.

Allogeneic CAR-exosomes carry donor HLA molecules, potentially triggering immune rejection, complement activation, or the formation of anti-HLA antibodies [286]. Repeated dosing may induce anti-exosome antibodies targeting surface proteins, reducing therapeutic efficacy and potentially causing hypersensitivity reactions. Furthermore, exosomal phosphatidylserine (PS) exposure may trigger coagulation cascades in some contexts [287,288].

## 5. Conclusions

CAR-derived exosomes represent a significant evolution in cancer immunotherapy, offering a cell-free platform that addresses certain limitations of living CAR cell therapies. Their nanoscale size, non-replicative nature, and potential for off-the-shelf production provide advantages in safety, tissue penetration, and manufacturing scalability. Preclinical studies demonstrate antitumor activity against hematologic malignancies and solid tumors, with favorable toxicity profiles compared to CAR-T cells. However, critical challenges temper enthusiasm for their rapid clinical translation. CAR-exosomes’ inability to expand in vivo fundamentally limits their durability, necessitating repeated administration and potentially restricting their efficacy in settings requiring long-term immune surveillance. Suboptimal tumor accumulation due to hepatosplenic clearance, variable cargo compositions complicating manufacturing standardization, and the incomplete understanding of tumor microenvironment interactions represent substantial translational barriers. Comparative analyses reveal that CAR-exosomes typically exhibit 30–60% of parent CAR-T cell cytotoxic potency, with activity persisting for days rather than months.

Realistic clinical translation will likely position CAR-exosomes not as replacements for CAR-T cells, but as complementary tools tailored to specific contexts: maintenance therapy following CAR-T or chemotherapy, treatment of patients ineligible for live cell therapies, or combination approaches where transient, targeted cytotoxicity is sufficient. With continued innovations in exosome engineering, manufacturing, and clinical trial design, CAR-exosomes may fulfill their promise as safer, scalable components of multi-modal cancer immunotherapy regimens.

## Figures and Tables

**Table 1:** Summary of CAR engineering methods, highlighting their mechanisms, advantages, and limitations.

Method	Description	Advantages	Limitations
Retroviral Vectors	Use of γ-retroviruses or lentiviruses to stably integrate CAR transgene into host genome via reverse transcription.	Provides stable, long-term expression; well-established and efficient in T cells.	Risk of insertional mutagenesis; lower transduction efficiency in NK cells; biosafety concerns.
Transposons	Mobile DNA elements (e.g., Sleeping Beauty, PiggyBac) that integrate CAR transgene into genome via DNA transposition.	Large cargo capacity; high transduction efficiency; low immunogenicity; cost-effective.	Risk of insertional mutagenesis; potential oncogenic transformation; variation in integration sites.
CRISPR/Cas9	Gene editing to knock-in CAR transgene precisely into specific genomic loci using homology-directed repair.	Precise integration; can target safe harbor loci; enables simultaneous gene knock-out/knock-in.	Limited cargo capacity; potential genomic instability; complex optimization needed.
CAR-mRNA Transfection	Transient expression via electroporation or lipid nanoparticle delivery of mRNA encoding CARs into cells.	Reduced risk of insertional mutagenesis/toxicity; fast expression onset; flexible dosing.	Transient; requires repeated administration; variable expression duration; cytotoxicity with electroporation.

**Table 2:** Post-translational modifications of proteins that are secreted in EVs.

Post-Translational Modification	Chemical Modification	Mechanism
Phosphorylation	Attachment of a phosphoryl group via kinases	Phosphorylation alters protein–protein interactions and membrane association, favoring inclusion into intraluminal vesicles [130,137]. Arf6 phosphorylation via ERK pathway activates phospholipase D2, inducing negative membrane curvature [138]. N-terminal phosphorylation of cargo proteins favors interactions with lipid rafts [139].
Ubiquitylation	Attachment of ubiquitin molecules via E1–3 ligase family	Membrane-anchored ubiquitin-like protein promotes sorting of proteins into small extracellular vesicles [131]. ESCRT 0-II proteins contain ubiquitin recognition domains (URDs), allowing incorporation into EVs [132].
SUMOylation	Addition of small ubiquitin-like modifier (SUMO) proteins via E1-E3 ligase family	SUMO modification may modulate protein interactions and localization relevant to EV loading [130]. SUMO-2 binds proteins through a SUMO interaction motif located between an α-helix and β-sheet of SUMO-2, particularly at amino acids Q30, F31, and I33. TSG101 has a putative SIM domain that sorts SUMO-2 into extracellular vesicles through ESCRT. SUMO-2 also interacts with negatively charged domains including phosphoinositols [140].
N-Glycosylation	Covalent attachment of glycans to asparagine residues	Glycosylated EV proteins are enriched in mannose, polylactosamine, α-2,6-sialic acid, and complex N-linked glycans [141]. Glycan scaffolds influence trafficking, receptor–lectin interactions, and protein stability, with subsequent internalization into EVs [130,137].
Palmitoylation	Covalent attachment of palmitoyl to cysteine, serine, or threonine residues	Palmitoylation allows protein attachment and incorporation into extracellular vesicles (EVs). Palmitoylation maintains protein conformation, protects against proteolytic degradation, and allows interactions with other membrane-bound proteins [142].
ISGylation	Covalent attachment of ISG15 protein by an isopeptide bond	ISG15 conjugation of the ESCRT component TSG101 induces aggregation and lysosomal degradation, reducing MVB number and impairing exosome secretion [134].

## Data Availability

No new data were created or analyzed in this study.

## Abbreviations

The following abbreviations are used in this manuscript:

AKT	Protein Kinase B
APC	Antigen Presenting Cell
ATP	Adenosine Triphosphate
CAR	Chimeric Antigen Receptor
CRISPR	Clustered Regularly Interspaced Short Palindromic Repeats
CRS	Cytokine Release Syndrome
CSF	Colony Stimulating Factor
DAG	Diacylglycerol
DC	Dendritic Cell
DNA	Deoxyribonucleic Acid
DNMT	DNA Methyltransferase
ECM	Extracellular Matrix
EPR	Enhanced Permeability and Retention
ERK	Extracellular Signal Regulated Kinase
ESCRT	Endosomal Sorting Complex Required for Transport
EV	Extracellular Vesicle
FDA	Food and Drug Administration
GARS1	Glycyl-tRNA Synthetase 1
GDP	Guanosine Diphosphate
GM-CSF	Granulocyte-Monocyte Colony Stimulating Factor
GMP	Good Manufacturing Practice
GTP	Guanosine Triphosphate
HDR	Homology-Directed Repair
HIF	Hypoxia- Inducible Factor
HLA	Human Leukocyte Antigen
ICANS	Immune Effector Cell-Associated Neurotoxicity Syndrome
IFN	Interferon
IL	Interleukin
ILV	Intraluminal Vesicle
LAT	Linker for Activation of T cells
LTR	Long Terminal Repeat
MEK	Mitogen-Activated Protein Kinase
MHC	Major Histocompatibility Complex
miRNA	MicroRNA
mRNA	Messenger RNA
MSLN	Mesothelin
MVB	Multivesicular Body
NF-κB	Nuclear Factor Kappa B
NFAT	Nuclear Factor of Activated T Cell
NHEJ	Non-Homologous End Joining
NK	Natural Killer
PA	Phosphatidic Acid
PB	PiggyBac
PD-1	Programmed Cell Death Protein 1
PD-L1	Programmed Death Ligand 1
PEG	Polyethylene Glycol
RNA	Ribonucleic Acid
ROS	Reactive Oxygen Species
SAP	SLAM-Associated Protein
SB	Sleeping Beauty
SEC	Size Exclusion Chromatography
siRNA	Small Interfering RNA
SNARE	Soluble N-ethylmaleimide-sensitive Factor Attachment Protein Receptor
SUMO	Small Ubiquitin-Like Modifier
TCR	T Cell Receptor
TFF	Tangential Flow Filtration
TGF-β	Transforming Growth Factor β
TIGIT	T Cell Immunoreceptor with Ig and ITIM Domains
TLR	Toll-Like Receptor
TME	Tumor Microenvironment
TNF-α	Tumor Necrosis Factor α
UC	Ultracentrifugation
VEGF	Vascular Endothelial Growth Factor
